# Quinazolines [*a*]-Annelated by Five-Membered Heterocycles: Synthesis and Biological Activity

**DOI:** 10.3390/molecules30173506

**Published:** 2025-08-27

**Authors:** Galina N. Lipunova, Emiliya V. Nosova, Valery N. Charushin

**Affiliations:** 1 Postovsky Institute of Organic Synthesis, Ural Branch of the Russian Academy of Sciences, 22 S. Kovalevskaya St./20 Akademicheskaya St., Ekaterinburg 620137, Russia; lipunova@ios.uran.ru (G.N.L.); charushin@ios.uran.ru (V.N.C.); 2 Department of Organic and Biomolecular Chemistry, Ural Federal University, 19 Mira St., Ekaterinburg 620002, Russia

**Keywords:** pyrroloquinazolines, indoloquinazolines, azoloquinazolines, biological activity, advanced synthesis

## Abstract

This review covers article and patent data obtained mostly within the period 2013–2024 on the synthesis and biological activity of quinazolines [*a*]-annelated by five-membered heterocycles. Pyrrolo-, (iso)indolo-, pyrazolo-, indazolo-, (benz)imidazo-, (benz)thiazolo-, and triazolo- [*a*]quinazoline systems have shown multiple potential activities against numerous targets. We highlight that most research efforts are directed to design of anticancer, antibacterial, anti-inflammatory, and other agents of azolo[*a*]quinazoline nature. This review emphases both the medicinal chemistry aspects of pyrrolo[*a*]-, (iso)indolo[*a*]-, and azolo[*a*]quinazolines and the comprehensive synthetic strategies of quinazolines annelated at the N(1)–C(2) bond from the perspective of drug development and discovery.

## 1. Introduction

Quinazoline and quinazolinone are medicinally important nitrogen heterocycles, exhibiting diverse biological activities [1,2]. The varied and complex effects that quinazoline compounds have on different biological systems have made them interesting for drug researchers who want to uncover all possible medical uses they might offer [3]. More than 150 naturally occurring alkaloid molecules contain the quinazolinone structure either by itself or within conjugated systems; most natural products of fused quinazoline origin are [*b*]-annelated quinazolines [4].

**Figure 1:**
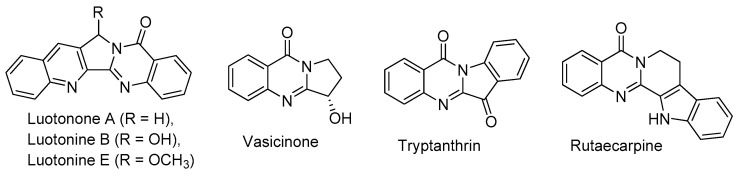
Polycyclic quinazolines of nature origin, which can be regarded as prototypes of biologically important [*a*]-annelated quinazolines.

Quinazolinone natural products display a wide range of medicinal properties, including antibacterial. One of them, Luotonin A (Figure 1), proved to exhibit significant cytotoxicity toward the murine leukemia P-388 cell line (IC_50_ = 1.8 μg/mL); this agent acts as a topoisomerase-I inhibitor [5]. Vasicinone (Figure 1), another natural alkaloid isolated from *P. nigellastrum*, is a potential agent for Parkinson’s disease and possibly other oxidative stress-related neurodegenerative disorders [4]. Indolo[*b*]quinazoline tryptanthrin (Figure 1) exhibits a broad spectrum of biological activities and can act as an anti-inflammatory, anticancer, antibacterial, antifungal, antiviral, and antitubercular agent, as well as other uses [4]. Rutaecarpine (Figure 1) is a naturally occurring pentacyclic indolopyridoquinazolinone alkaloid extracted from the traditional Chinese herb *Evodia rutaecarpa*. It has long been utilized in folk medicine for alleviating symptoms related to headaches, gastrointestinal disturbances, and various other illnesses. Building upon its medicinal potential, numerous therapeutic agents have since been developed targeting inflammation, atherosclerosis, Alzheimer’s disease, cancerous tumors, and fungal infections [4]. Considering the directions of quinazolines modification for potential use in medicinal chemistry, in some cases, chemists designed the angular counterparts of [*b*]-annelated quinazoline biologically active compounds.

Annelated quinazolines synthesized by various methods have gained significant attention from researchers worldwide due to their diverse and beneficial health-related characteristics [6,7]. The intensive utilization of the quinazoline core for designing and synthesizing novel antibacterial agents has recently received considerable attention. Review article [5], devoted to antibacterial activity of various 4(3*H*)-quinazolinone derivatives, discloses some data on thiazolo[3,4-*a*]quinazolinones active against *B. subtilis* and SAR studies on [1,2,4]-triazolo[*a*]quinazolines. Scientists combine parts from different active molecules, like quinazolines, using special techniques to create new hybrid compounds that work better and stronger [8].

The introduction of new approaches to the design of biologically active derivatives with improved properties stimulates the development of modern synthetic strategies, such as green chemistry techniques, one-pot processes, and other rational methods. One-pot synthesis procedures of quinazoline derivatives, emerging as a highly efficient and sustainable strategy for the preparation of novel bioactive compounds, are summarized in the review [9].

Quinazolines [*c*]-annelated by five- and six-membered heterocycles were analyzed recently [10]. Different triazoloquinazoline derivatives were overviewed [11], and it was stated that the condensation of 2-hydrazino-3*H*-quinazolin-4-ones with carbonic acids represents the most common synthetic approach to 1,2,4-triazolo[4,3-*a*]quinazolines, whereas their isomers, [1,2,4]triazolo[1,5-*a*]quinazolines, are obtained by cyclocondensation of *N*-cyanoimido(dithio)carbonates with 2-hydrazinobenzoic acid. A variety of biological applications of triazoloquinazolines and imidazoquinazolines are highlighted in the book chapters [12,13]. Synthesis and bioactivity evaluation of some pyrazolo[*c*]quinazolines were described in reviews [14,15].

The annelation of an additional ring at the N(1)–C(2) bond represents a crucial modification of quinazolines that enables fine-tuning of their biological activity. Consequently, [*a*]-annelated quinazolines attract substantial interest owing to their notable pharmacological properties. To date, compounds of this type have not been introduced into medical practice. Vaskevych et al. [16] provided the comprehensive analysis of quinazolin-4-one heteroanalogues as biologically relevant substances and drugs. They paid special attention to cyclizations of alkenyl(alkynyl)-functionalized quinazolinones into pyrrolo-, thiazolo-, and imidazo[*a*]- and [*b*]-annelated quinazolin-4-ones; structural and electronic effects of reagents on the regio- and stereoselectivity of the cyclizations were elucidated, and relevant reaction mechanisms were clarified.

**Figure 2:**
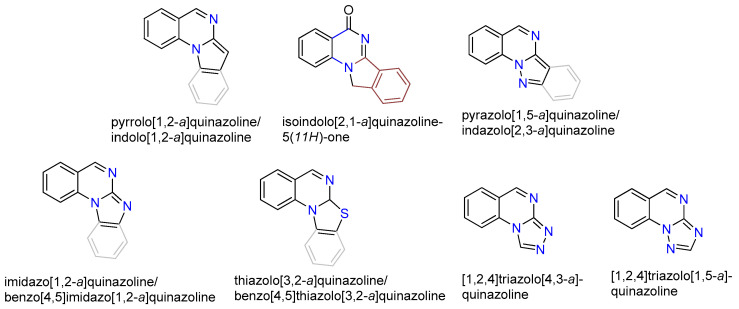
Types of polycyclic quinazolines considered in current review.

In this review, we focus on the medicinal chemistry aspects of various quinazolines [*a*]-annelated by five-membered heterocycles (Figure 2), encompassing both synthetic pathways and biological activities. We compile relevant literature reports within the context of drug development and discovery efforts.

A wide variety of methods are essential for constructing such annelated systems. Traditional methods for cyclocondensation may work well but also come with some drawbacks and limitations. Innovative syntheses utilizing appropriate reagents, multicomponent reactions, intramolecular C–H amidations, *N*-arylation/2-amidation cascades, along with metal-free, catalyst-free eco-friendly strategies, are becoming increasingly prevalent.

## 2. Pyrrolo[1,2-*a*]quinazolinones

In review article [17], one section focuses on pyrrolo[1,2-*a*]quinazolines, detailing their synthesis strategies and providing illustrative examples. Following this publication, numerous research teams began synthesizing similar compounds and evaluating their biological effects, with particular emphasis placed on structural derivatives of Luotonin and Vasicinone.

**Scheme 1:**
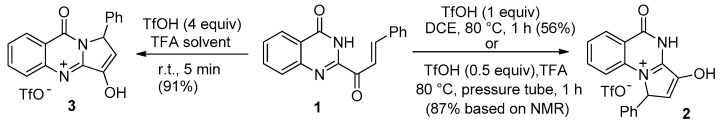
Synthetic approach to [*a*]- or [*b*]-annelated quinazolinones **2** and **3**.

Rasapalli et al. [18] investigated the cyclizations of quinazolinonyl enone **1**, using super acids, to access the C-aryl luotonins via the intermediacy of tricyclic ketones. It was found that depending on substoichiometric amounts of TfOH in TFA as a solvent the cyclization of quinazolinonyl enone **1** proceeds with the formation of [*a*]- or [*b*]-annelated quinazolinones **2** or **3** as triflate salts of the enol tautomers (Scheme 1). It was found that in a more acidic medium, compound **2** is capable of effective conversion to regioisomer **3**. Based on data from quantum chemical calculations, the probable mechanism for the formation of regioisomeric tricyclic enols was discussed, and it was suggested that the cyclization process follows an aza-Nazarov pathway.

**Scheme 2:**
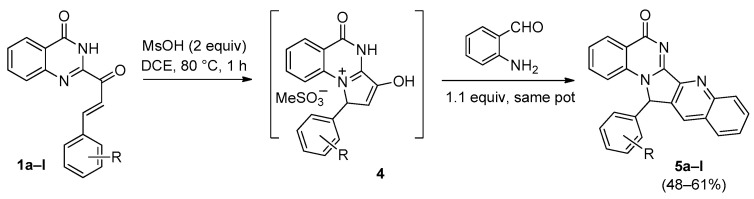
Synthesis of angular luotonins **5a**–**l**. **1**, **4**, **5**: R = H (**a**), 4-OMe (**b**), 4-Cl (**c**), 3-OMe (**d**), 3,4,5-(OMe)_3_ (**e**), 4-Br (**f**), 4-F (**g**), 4-CF_3_ (**h**), 3-Br (**i**), 4-SMe (**j**), 2-Me (**k**), 4-Me (**l**).

In subsequent work [19], the same team successfully developed a facile one-pot synthesis of novel angular luotonins **5a**–**l** via a methanesulfonic acid mediated aza-Nazarov–Friedlander cyclization sequence of quinazolinonyl enones **1a**–**l** (Scheme 2). Optimal conditions for the formation of tricyclic enols **4** with a yield of up to 59% were selected, and two-step synthesis of polycyclic compounds **5** was carried out without isolation of intermediates **4**. The structure of polycyclic compounds **5f** and **5k** was confirmed by X-ray data.

A series of angular luotonins **5a**–**l** were assayed as topo-I inhibitors at four different concentrations (0.1, 1.0, 10, and 100 μM), camptothecin (CPT, at 1 μM) was used as the standard. Pentacyclic derivatives **5** proved to be weak inhibitors of human topoisomerase I compared to CPT, despite their nearly planar structure [19], which can be effectively incorporated into specific binding sites on the target biomolecules.

**Scheme 3:**
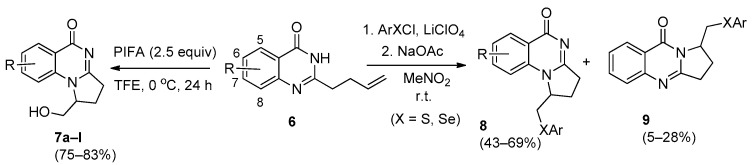
Synthesis of 2,3-dihydropyrrolo[1,2-*a*]quinazolin-5(1*H*)-ones **7**. **7**: R = H (**a**), 5-NO_2_ (**b**), 5-F (**c**), 6-Me (**d**), 6-OMe (**e**), 6-Cl (**f**), 6-NO_2_ (**g**), 6,7-(OMe)_2_ (**h**), 7-Cl (**i**), 8-Me (**j**), 8-Br (**k**), 8-F (**l**).

Vaskevych et al. [20] developed an efficient approach to 1-(hydroxymethyl)-2,3-dihydropyrrolo[1,2-*a*]quinazolin-5(1*H*)-ones **7**, the method is based on oxidative cyclization of 2-(3-butenyl)quinazolin-4(3*H*)-ones **6** initiated by iodosobenzene bis(trifluoroacetate) (PIFA) (Scheme 3). The reaction proceeds with high regioselectivity when 2.5 eq of PIFA are used in 2,2,2-trifluoroethanol (TFE) solution at 0 °C over 24 h. Quinazolinone derivatives **6** were obtained in good yields (63–91%) in two stages: acylation of anthranilamides with α-allylacetyl chloride and subsequent thermal cyclocondensation. The structure of compounds **7** was confirmed by spectral data, including X-ray for derivative **7f**. Compounds **7** represent a new class of vasicinone analogs.

Arylsulfenylation (selenylation) of 2-(3-butenyl)quinazolin-4(3*H*)-ones **6** in nitromethane in the presence of an equimolar amount of lithium perchlorate proceeds as an electrophilic cyclization involving N(1) or N(3) atoms of quinazolone core to yield 1-arylthio(selenyl)methyl substituted angular-annulated 2,3-dihydropyrrolo[1,2-*a*]quinazolin-5(1*H*)-ones **8** as major products and isomeric linear-annulated 2,3-dihydropyrrolo[2,1-*b*]quinazolin-9(1*H*)-one derivatives **9** as minor products (Scheme 3) [21].

**Scheme 4:**
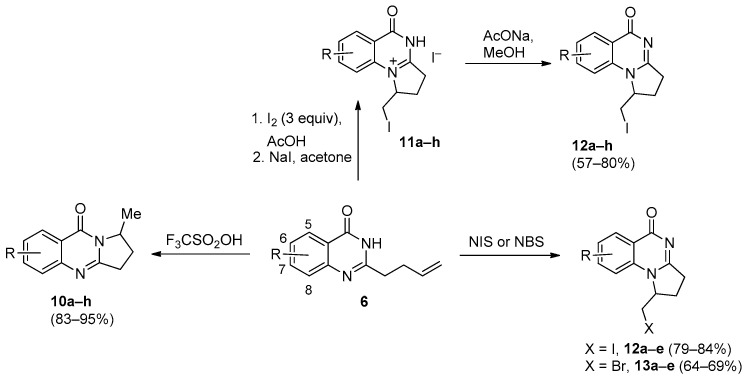
Synthetic approach to pyrroloquinazolinones **10**, **12**, **13**. R = H (**a**), 5-F (**b**), 6-Cl (**c**), 7-Cl (**d**), 8-Me (**e**), 6-OMe (**f**), 6-NO_2_ (**g**), 6,7-(OMe)_2_ (**h**).

Vaskevych et al. developed a selective synthetic approach to pyrroloquinazolinones based on the proton- and halogen-induced electrophilic cyclization of 2-(3-butenyl)quinazolin-4(3*H*)-ones **6** [22]. Several protic acids (HCl, CF_3_CO_2_H, H_2_SO_4_, polyphosphoric acid, CF_3_SO_3_H) were tested, and trifluoromethanesulfonic acid was chosen as the most efficient reagent. Compounds **6a**–**h** dissolved in CF_3_SO_3_H at room temperature were converted into 1-methyl-2,3-dihydropyrrolo[2,1-*b*]quinazolin-9(1*H*)-ones **10a**–**h** with high chemoselectivity and in excellent yields (Scheme 4). The linear structure was confirmed by XRD data using compound **10g** as an example.

Iodine, bromine, NIS, and NBS were used as halogenating agents in the synthesis of pyrrolo[1,2-*a*]quinazolinones **12** and **13**. The reaction of compounds **6a**–**h** with 3 equiv. of iodine in acetic acid followed by the treatment with sodium iodide led to pyrrolo[1,2-*a*]quinazolinonium salts **11a**–**h**, which when treated with sodium acetate gave 1-iodomethyl-substituted pyrrolo[1,2-*a*]quinazolinones **12a**–**h** in an overall yield of up to 80% (Scheme 4). The selective cyclization of compounds **6** using bromine failed due to the formation of bromination products at the double bond of quinazolinones **6**. The iodination of substrates **6a**–**e** with 1 eq of NIS in acetonitrile solution at 0 °C allows one-step preparation of target products **12a**–**e** in high yields. The treatment of **6a**–**e** with NBS under the same conditions led to the formation of 1-bromomethyl-substituted pyrrolo[1,2-*a*]quinazolinones **13a**–**e**.

**Scheme 5:**
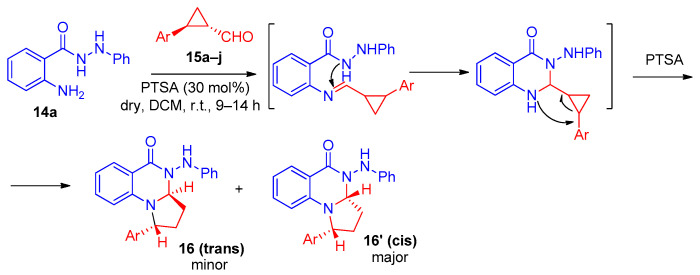
Synthesis of tetrahydropyrrolo[1,2-*a*]quinazolin-5(1*H*)-ones **16aa**–**ja** from **14a** and **15a**–**j**. **16aa**: Ar = 4-MeOC_6_H_4_, 85%, 34:66; **16ba**: Ar = Ph, 83%, 35:65; **16ca**: Ar = 4-MeC_6_H_4_, 82%, 32:68; **16da**: Ar = 3,4-(MeO)_2_C_6_H_3_, 81%, 40:60; **16ea**: Ar = 4-FC_6_H_4_, 82%, 35:65; **16fa**: Ar = 4-ClC_6_H_4_, 82%, 35:65; **16ga**: Ar = 4-BrC_6_H_4_, 81%, 35:65; **16ha**: Ar = 1-naphthyl, 78%, 36:64; **16ia**: Ar = styryl, 79%, 35:65; **16ja**: Ar = 2-furyl, 82%, 35:65.

Singh et al. developed a highly regioselective synthesis of tetrahydropyrrolo[1,2-*a*]quinazolin-5(1*H*)-one derivatives **16**, using such building blocks as *N*′-aryl benzohydrazides **14** and cyclopropane aldehydes **15** [23]. Optimal conditions for the annulation of *N*′-phenyl-2-aminobenzohydrazide **14a** with aldehydes **15** were found. The process was realized in DCM in the presence of PTSA (30 mol%) as a catalyst at room temperature. Presented easily scaled reaction follows a domino sequence of imination/intramolecular cycling/nucleophilic ring opening. The transformation proceeds via the formation of an intermediate imine species, which subsequently engages in intramolecular cyclization. The resulting dihydroquinazolin-4-one then experiences nucleophilic ring opening, followed by cyclization involving the nitrogen atom of the quinazoline ring. Interaction of differentially substituted cyclopropane aldehydes **15a**–**j** with *N*-phenylbenzohydrazide **14a** yielded a series of tetrahydropyrrolo[1,2-*a*]quinazolin-5(1*H*)-ones **16aa**–**ja** as two diastereoisomers (trans and cis) in an overall yield of 78–85% (Scheme 5).

**Scheme 6:**
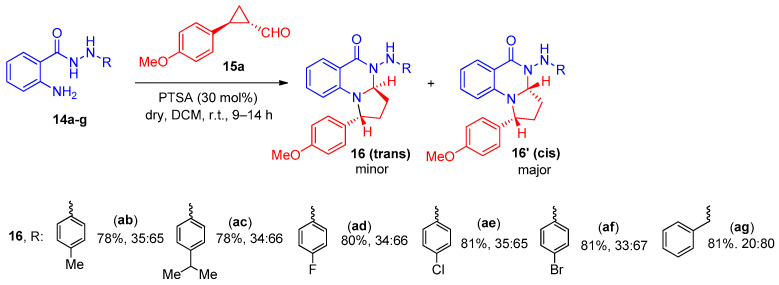
Synthesis of tetrahydropyrrolo[1,2-*a*]quinazolin-5(1*H*)-ones **16ab**–**ag** from **14a**–**g** and **15a**.

The universality of the reaction involving *N*’-aryl-substituted anthranyl hydrazides **14a**–**f** was studied by their interaction with 4-methoxyphenyl-cyclopropane aldehyde **15a** [23]. The formation of tetrahydropyrrolo[1,2-*a*]quinazolin-5(*1H*)-ones **16ab**–**ag** was also shown as *trans* and *cis* isomers with the same ratio, except **16ag**, and an overall yield of 78–81% (Scheme 6). It should be noted that *N*-benzyl anthranilamide and *N*-phenyl anthranilamide participate in the reaction with **15a**, which also led to the formation of tetrahydropyrrolo[1,2-*a*]quinazolin-5(*1H*)-ones in good yields. The relative configuration of both the diastereomers (cis and trans) was established based on the nuclear Overhauser effect (NOE) experiment data of **16aa** and **16aa′** and was supported by the X-ray analysis of both diastereomers **16ag**.

**Scheme 7:**
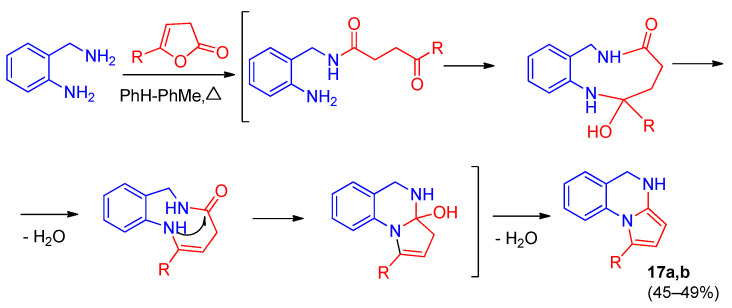
Synthesis of 1-R-4,5-dihydropyrrolo[1,2-*a*]quinazolines **17** from 2-(aminomethyl)aniline and furanones. R = Ph (**a**), 4-MeC_6_H_4_ (**b**).

Grinev et al. described the preparation of 1-R-4,5-didihydropyrrolo[1,2-*a*]quinazolines **17a**,**b** by the reaction of 2-(aminomethyl)aniline with furanones at heating in aprotic solvents (benzene, toluene) with azeotropic removal of eliminated water (Scheme 7) [24].

## 3. Indolo[1,2-*a*]quinazolinones and Isoindolo[2,1-*a*]quinazolinediones

Indoles are widely found in pharmaceuticals and nature products; the indole-containing quinazoline derivatives were reported as protein kinase CK2 inhibitors and poly(ADP-ribose)polymerase-1 (PARP-1) inhibitors [25]. Isoindoloquinazolines have been shown to be Tumor Necrosis Factor-alpha (TNF-*α*) inhibitory [26].

**Scheme 8:**
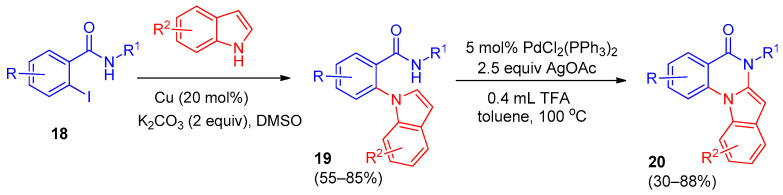
Synthetic approach to indolo[1,2-*a*]quinazolinone derivatives **20** from 2-iodobenzamides **18** and indoles. R = H, Cl, (MeO)_2_; R^1^ = Me, Et, iso-Pr, Bn, Ph, Ar; R^2^ = H, OMe, OBn, Cl, Br.

Synthetic approaches to bioactive indolo[1,2-*a*]quinazolinones are limited. Kotipalli et al. developed an easy and convenient two-step method for the synthesis of indolo[1,2-*a*]quinazolinone derivatives, starting from 2-iodobenzamides **18** and indole derivatives [27]. The first step involves the *N*-arylation of indole by 2-iodobenzamide derivatives via Ullman coupling. Optimal conditions for this stage were found, which allowed obtaining 2-(1*H*-indol-1-yl)-*N*-substituted benzamide derivatives **19** in up to 85% yield. The second step involves intramolecular C–H amidation using a palladium catalyst and AgOAc as oxidant and proceeds in toluene at 100 °C in the presence of TFA (Scheme 8). The series of indolo[1,2-*a*]quinazolinone derivatives **20** with different substituents R, R^1^, R^2^ was obtained. It was shown that long alkyl chain in R^1^ or the *p*-methoxy group in the aryl fragment leads to a decrease in yield to 30–34%; in the case R = NO_2_, the cyclization does not occur.

**Scheme 9:**
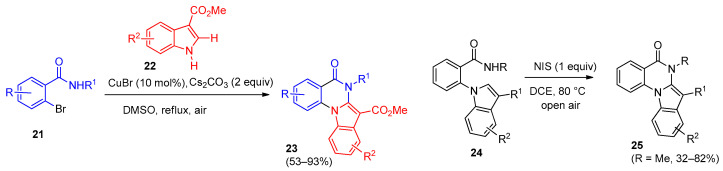
Synthesis of indolo[1,2-*a*]quinazolinones **23** and **25** from 2-bromobenzamides **21** and indole derivatives **22**. **23**: R = H, MeO, Cl, Br; R^1^ = H, Me, Bn, Ph; R^2^ = H, Me, Cl; **25**: R = H, Me, Et, Bn, Ar; R^1^ = H, Me; R^2^ = H, Me, Et, MeO, Cl, Br.

Abe et al. reported a self-relay copper (I)-catalyzed Ullmann *N-*arylation/2-amidation cascade to form functionalized indolo[1,2-*a*]quinazolinones **23** in one-pot from easily available 2-bromobenzamides **21** with indoles **22** in high yields [28] (Scheme 9). It was found that the optimal conditions for the cascade are the following: the presence of CO_2_Me group at the position 3 of indole, the use of CuBr as a catalyst, Cs_2_CO_3_ as a base and DMSO as a solvent, boiling for 16 h. It was also shown that in the case R = NO_2_ or R^1^ = *t*-Bu indolo[1,2-*a*]quinazolinones could not be obtained even under boiling for 72 h. The authors discuss the proposed process pathways and note that methyl carboxylate could act as activating group in this Ullmann *N*-arylation/2-amidation cascade.

Badigenchala et al. developed a transition-metal- and base-free approach to indolo[1,2-*a*]quinazolinones [29]. It was demonstrated that *N*-iodosuccinimide (NIS) can be used for intramolecular cross-coupling of C(sp^2^)–H and N–H bonds, leading to the formation of indolo[1,2-*a*]quinazolinones **25** (Scheme 9). Optimal cyclization conditions were found for 2-(1*H*-indol-1-yl)-*N*-methylbenzamide, and the effect of substituents R, R^1^, R^2^ on reaction ability was studied. It was shown that the presence of strong electron withdrawing (R^2^ = 5-NO_2_, 7-azaindole derived) or bulky (R = *t*-Bu) group suppressed the reaction completely. Gram scale synthesis has been performed to examine the synthetic utility of the protocol.

**Scheme 10:**
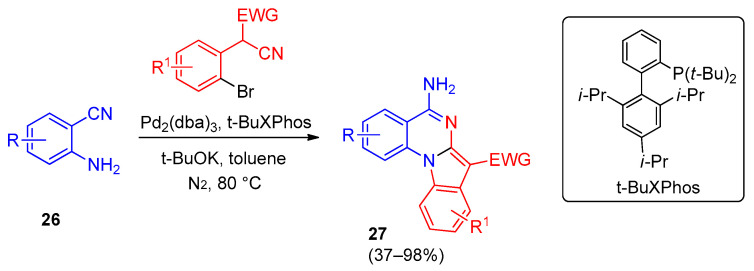
Synthesis of 5-amine-indolo[1,2-*a*]quinazolines **27** from 2-aminobenzonitriles **26** and 2-(2-bromophenyl)acetonitriles. R = H, Me, F, CF_3_; R^1^ = H, F, MeO; EWG = CN, COOEt.

Jiang et al. described Pd-catalyzed domino synthesis of 5-amine-indolo[1,2-*a*]quinazolines **27** from readily available 2-aminobenzonitriles **26** and 2-(2-bromophenyl)acetonitriles (Scheme 10) [30]. The approach involves a Buchwald–Hartwig type coupling and a base-promoted intramolecular nucleophilic reaction. It was found that in the case of EWG = COOEt, the yield of products **27** is reduced to 55–62%, and in the case of R = CF_3_, to 37%.

**Scheme 11:**
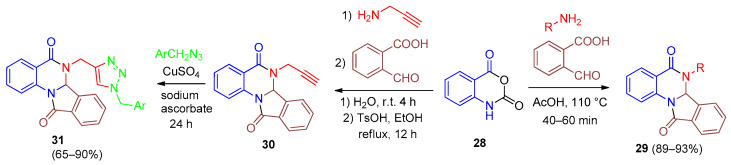
Synthetic approach to 6,6a-dihydroisoindolo[2,1-*a*]quinazoline-5,11-dione derivatives **29**, **31**. **29**: R = H, Me, Et, *n*-Pr, (CH_2_)_2_-OH, (CH_2_)_3_-OH, (CH_2_)_3_-COOH; **31**: Ar = Ph, 2-Me-C_6_H_4_, 4-F-C_6_H_4_, 2-Cl-C_6_H_4_, 2-Br-C_6_H_4_, 2-NO_2_-C_6_H_4_, 2,3-Cl_2_-C_6_H_3_, 3,4-Cl_2_-C_6_H_3_.

One approach to the synthesis of isoindoloquinazolinediones is based on a multi-component reaction (MCR) of isatoic anhydride **28**, amines and 2-formylbenzoic acid under different conditions (Scheme 11). The reaction was carried out by heating the reagents in acetic acid at 110 °C, aliphatic and aromatic amines were used, the highest yields of 6,6a-dihydroisoindolo[2,1-*a*]quinazoline-5,11-diones **29** were obtained for the more nucleophilic aliphatic amines [31]. Reddy et al. [32] described the faster and greener synthesis of the analogues of compounds **29** via a β-cyclodextrin-mediated MCR of the same reagents in water under microwave irradiation.

Esmaeili-Marandi et al. [33] described novel 1,2,3-triazole-containing isoindolo[2,1-*a*]-quinazolines via a convenient three-step reaction starting from isatoic anhydride **28**. Interaction of **28** with 1-aminoprop-2-yne in water at room temperature gives 2-amino-*N*-(prop-2-yn-1-yl)benzamide, which further undergoes cyclization into isoindolo[2,1-*a*]-quinazoline **30** in the presence of TsOH (20 mol%) in EtOH under reflux (Scheme 11). Product **30** was readily converted to the target compound **31** via click reaction with organic azides, obtained from the corresponding benzyl halogenides and sodium azide.

**Scheme 12:**
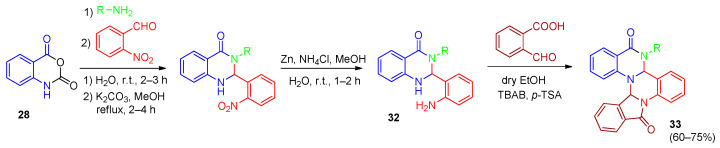
Synthetic approach to isoinodolo[2,1-*a*]quinazolino[1,2-*c*]quinazolinones **33**. R = (CH_2_)_2_Ph, cyclopentyl, cyclopropyl, allyl, hexyl, butyl, propyl, ethyl.

Mahdavi et al. developed a four-step effective and simple synthetic approach for the synthesis of novel isoinodolo[2,1-*a*]quinazolino[1,2-*c*]quinazolinones **33** (Scheme 12) [34], the method can be extended for the preparation of a library of potentially bioactive compounds. Reaction of isatoic anhydride **28** and amines led to various 2-aminobenzamide derivatives, and their interaction with *o*-nitrobenzaldehyde and subsequent reduction of nitro group resulted in the formation of 2-(2-aminophenyl)-3-R-2,3-dihydroquinazolin-4*(*1*H)*-ones **32**. Optimal conditions for the reaction of compounds **32** with 2-formylbenzoic acid were found: boiling in dry ethanol in the presence of *p*-toluenesulfonic acid (*p*-TSA) and tetrabutylammonium bromide (TBAB) at a ratio of *p*-TSA/TBAB = 0.5 [34].

**Scheme 13:**
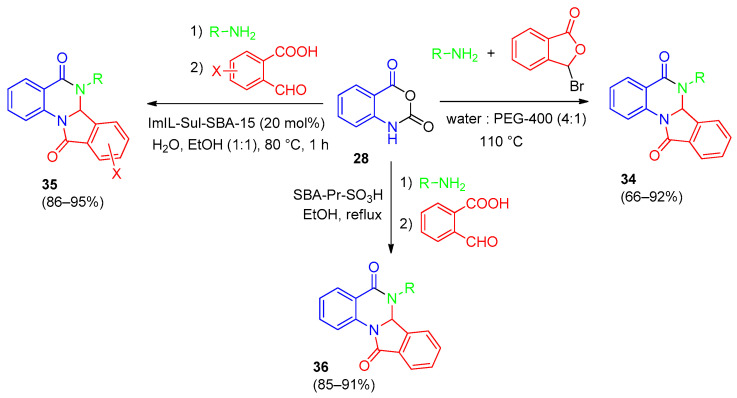
Synthesis of dihydroisoindolo[2,1-*a*]quinazoline-5,11-dione derivatives **34**, **35**, **36**. **34**: R = Me, Bu, prop-2-yne, cyclopropyl, (CH_2_)_2_Ph, 1-Nf, Bn-4-Cl, Bn-4-OMe, Bn-2,4-Cl_2_, 4-Me-C_6_H_4_, 4-CN-C_6_H_4_, 2-MeO-C_6_H_4_, 2,3-(Me)_2_-C_6_H_3_, 2-Cl,3-Me-C_6_H_3_, 4-COOMe-C_6_H_4_, 4-*t*-Bu-C_6_H_4_; **35**: R = propen-2-yl-methyl, Bn, Bn-2-Cl, Bn-4-Cl, furan-2-yl-methyl, Py-2-yl-methyl; X = H, 2,3-(MeO)_2_; **36**: R = propen-2-yl, *n*-Pr, *n*-Bu, cyclopropyl, Bn, Bn-4-Me, Bn-2-OMe, Ph, 4-Me-C_6_H_4_, 4-MeO-C_6_H_4_.

Recently, performing metal-catalyst-free green and efficient synthesis in water has become more popular because it is good for the environment and sustainable. Madhubabu et al. [35] developed a short and efficient metal free, catalyst-free greener approach for the synthesis of dihydroisoindolo[2,1-*a*]quinazoline-5,11-dione derivatives **34** in water (Scheme 13). Isatoic anhydride, an amine, and 3-bromoisobenzofuran-1(3*H*)-one served as starting materials. A mixture of water and polyethylene glycol 400 (PEG-400) in a ratio of 4:1 provided the most efficient solvent system. After completing the reaction, the product underwent filtration, and the recovered solvent could be effectively reused multiple times without noticeable loss of activity compared to fresh solvent. This reliable methodology tolerates both aliphatic and aromatic amines and exhibits exceptional scalability.

Abbasian et al. [36] reported the synthesis of dihydroisoindolo[2,1-*a*]quinazoline-5,11-diones **35** using the catalyst on silica-based ordered mesoporous material (SBA-15) functionalized by imidazolium ionic liquid sulfonic acid (ImIL-Sul-SBA-15) (Scheme 13). The reaction between isatoic anhydride **28**, amines, and substituted 2-formylbenzoic acids was conducted in the ethanol–water mixture (1:1) in the presence of ImIL-Sul-SBA-15 (20 mol%) at 50 °C for 1 h. Once the reaction finished, a mixture containing desired product **35** along with the catalyst (ImIL-Sul-SBA-15) was filtered out. Then, this solid was cleaned with ethanol to remove the product, leaving behind just the catalyst. Testing demonstrated that the catalyst maintained its efficiency and could be recycled up to six consecutive cycles without experiencing any noticeable loss in catalytic activity. Benefits include easy recovery of the catalyst, quick reaction time, and high yields of the final product.

Rayatzadeh et al. described the synthesis of a series of 6,6*a*-dihydroisoindolo[2,1-*a*]quinazoline-5,11-dione derivatives **36** using sulfonic acid functionalized nanoporous silica (SBA-Pr-SO_3_H) as a reusable catalyst (Scheme 13) [37]. Derivatives **36** were obtained via a two-step process, once the reaction was completed, the catalyst was separated, and the final products were isolated by recrystallization from ethanol. Key advantages are high yields and easy purification process.

**Scheme 14:**
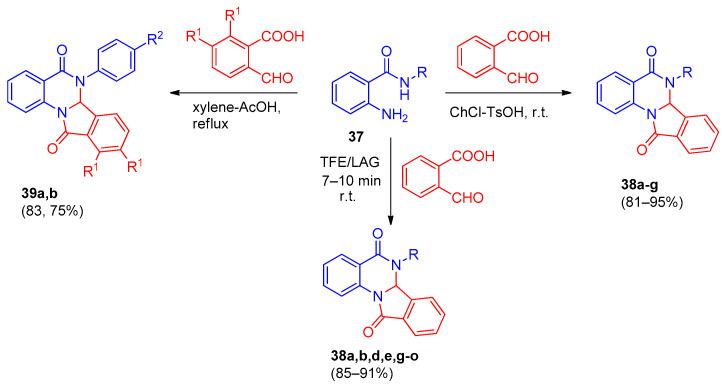
Synthetic approach to dihydroisoindolo[2,1-*a*]quinazolin-5,11-diones **38**, **39** from 2-aminobenzamides **37** (in blue) and 2-formylbenzoic acids (in red). **37**: R = H, Alk, Bn, Ar; **38**: R = H (**a**), Bn (**b**), Me (**c**), Ph (**d**), 4-Me-C_6_H_4_ (**e**), 3-Me-C_6_H_4_ (**f**), 4-MeO-C_6_H_4_ (**g**), 4-ClC_6_H_4_ (**h**), 4-BrC_6_H_4_ (**i**), 4-NO_2_C_6_H_4_ (**k**), 3-NO_2_C_6_H_4_ (**l**), 2-Nf (**m**), cyclohexyl (**n**), Bu (**o**); **39**: R^2^ = R^1^ = H (**a**), R^2^ = R^1^ = OMe (**b**).

The synthesis of isoindoloquinazolinediones **38** was carried out in a single step starting from 2-aminobenzamides **37** and 2-formylbenzoic acid, conditions for cyclization were selected (Scheme 14) [38,39,40]. Devi et al. [38] used a mixture of choline chloride (ChCl) and TsOH as promising green deep eutectic solvent. The reaction proceeds rapidly (15 min) at room temperature leading to dihydroisoindolo[2,1-*a*]quinazolin-5,11-diones **38a**–**g** in high yields.

Lohar et al. developed an efficient and green mechanochemical method for the synthesis of isoindolo[2,1-*a*]quinazolines **38a**,**b**,**d**,**e**,**g**–**o** via 2,2,2-trifluoroethanol (TFE)-catalyzed liquid-assisted grinding (LAG) (Scheme 14) [40]. A mixture of equimolar amounts of reagents and TFE was ground together for 9–11 min at room temperature; the progress of the reaction was monitored by TLC, compounds **38a**,**b**,**d**,**e**,**g**–**o** were isolated in high yields.

Dihydroisoindolo[2,1-*a*]quinazolin-5,11-diones **39** were prepared by cyclization in the xylene/acetic acid mixture at reflux for 3–5 h and isolated as racemates (Scheme 14). The authors of [39] performed a prediction of biological activity profile of compound **39a** and concluded that this quinazolinone derivative can affect many molecular targets within cells, promising for design new anticancer and antiparasitic drugs, as well as agents for neurodegenerative diseases therapy. Thus, 6*aS*-enantiomer of annelated quinazoline **39a** can block the TDP1 and ELG1 protein, suppress the ATG4B protease function. The racemic mixture of the tetracyclic compound **39a** is toxic for cardiomyocytes, it can decompose the TDP-43 protein, inhibit the DNA repair systems.

**Scheme 15:**
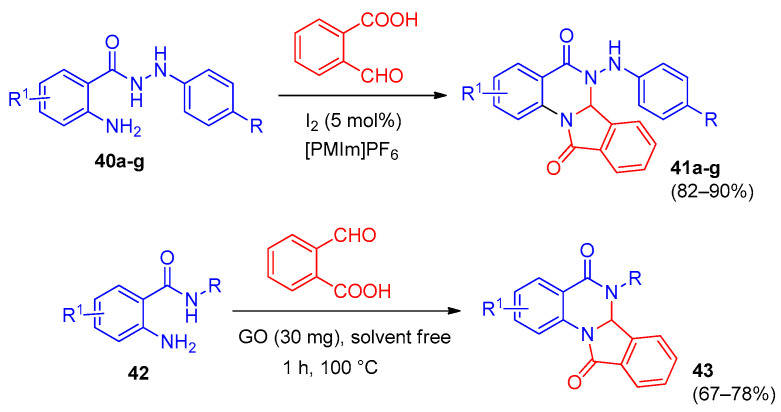
Synthetic approach to dihydroisoindolo[2,1-*a*]quinazoline-5,11-diones **41**, **43**. **41**: R, R^1^ = Me, 5-Br (**a**); H, 5-Me (**b**); Me, H (**c**); Cl, 5-Br (**d**); H, H (**e**); H, 5-Cl (**f**); Me, 4-Cl (**g**); **43**: R = NHAr, CH_2_Ar, allyl, cyclopropyl, etc.; R^1^ = H, Br, Cl.

A series of 6-(arylamino)-6,6*a*-dihydroisoindolo[2,1-*a*]quinazolin-5,11-diones **41a**–**g** was synthesized through iodine-catalyzed reaction of *N*’-aryl-2-aminobenzohydrazides **40** and 2-formylbenzoic acid in ionic liquid (Scheme 15) [41]. The structure of the compounds was confirmed by X-ray data, for compound **41b** as an example. The reaction was shown to be chemoselective, the interaction of unsubstituted 2-aminobenzohydrazide with 2-formylbenzoic acid under the same conditions gave 5*H*-phthalazino[1,2-*b*]quinazoline.

A fast and eco-friendly technique was created to prepare a series of important, versatile isoindolo[2,1-*a*]quinazolines **43** with multiple functions for biology research (Scheme 15) [42]. The reaction of 2-aminobenzhydrazide **42** (R = H) with 2-formylbenzoic acid was studied under solvent free conditions using graphene oxide (GO) as mild heterogeneous carbocatalyst. Optimal conditions have been identified, expanding the scope of 2-aminobenzohydrazides. It has been demonstrated that 2-aminobenzamides give high yield of products **43** under the same conditions. The structures of compounds **43** have been validated through X-ray analysis for two compounds **43** (R = NHPh, R^1^ = H, and R = CH_2_C_6_H_4_-4-Me, R^1^ = 3-Cl). The GO nanosheets were prepared from natural graphite powder. A possible reaction mechanism with GO as catalyst was discussed, it was noted that the acidity of GO plays an important role in the studied transformation.

**Scheme 16:**
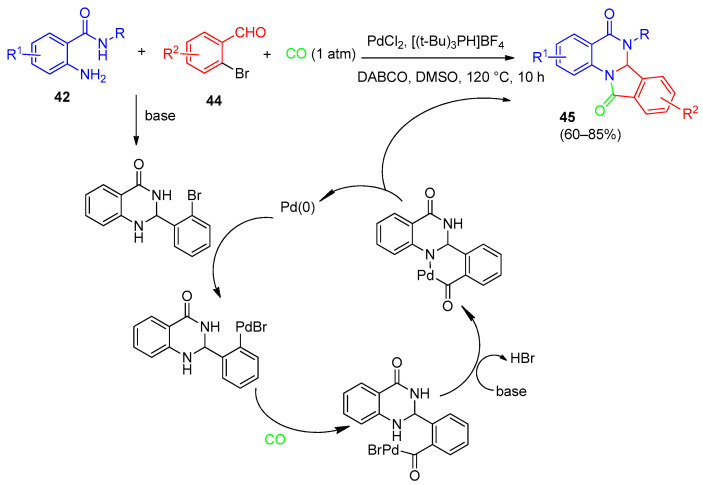
Synthesis of dihydroisoindolo[2,1-*a*]quinazoline-5,11-dione derivatives **45**. **42**, **45**: R = H, Bn, NHPh; R^1^ = Me, MeO, Cl; **44**, **45**: R^2^ = Me, OMe, F, Cl, CF_3_.

Guo et al. [43] reported the synthesis of dihydroisoindolo[2,1-*a*]quinazolin-5,11-diones **45** without using 2-formylbenzoic acid. The preparation process includes palladium-catalyzed three-component carbonylative cyclization of 2-aminobenzamides **42** with 2-bromobenzaldehydes **44** under an atmospheric pressure of carbon monoxide (Scheme 16). Optimal synthetic conditions, including selection of catalyst, ligand, base, and solvent, were found, and broad substrate scope was demonstrated. A plausible mechanism involves a palladium-catalyzed cyclocondensation/cyclocarbonylation sequence: initially, the oxidative addition of Pd(0) to 2-(2-bromophenyl)tatrehydroquinazolin-4-one, which is formed in situ via the cyclocondensation of 2-aminobenzamide **42** with 2-bromobenzaldehyde **44** under basic conditions, gives rise to a palladium complex. Next, the insertion of CO into the C–Pd bond of this complex affords an acylpalladium complex, which then eliminates HBr regioselectively to yield cyclopalladium intermediate under the promotion of the base. Finally, reductive elimination delivers **45** and regenerates the Pd(0) species. It is also possible that the intramolecular nucleophilic attack of NH on the carbonyl group of acylpalladium complex gives product **45** and the active Pd(0) species. According to Guo et al. [43], this method works well with different types of chemical groups, uses easily available ingredients, demonstrates high regioselectivity, and is straightforward to perform.

**Scheme 17:**
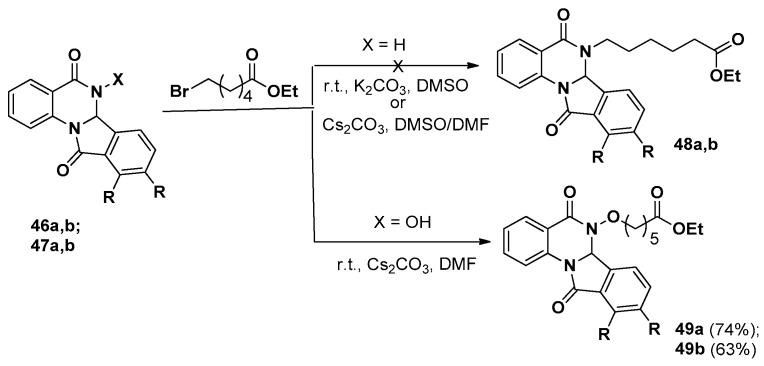
Synthetic approach to dihydroisoindolo[2,1-*a*]quinazoline-5,11-diones **49a**,**b**. **46**: X = H, R = H (**a**), OMe (**b**); **47**: X = OH, R = H (**a**), OMe (**b**); **49**: R = H (**a**), OMe (**b**).

Kolotaev et al. [44] described synthetic approaches to dihydroisoindolo[2,1-*a*]quinazolin-5,11-diones, containing hydroxamic acids, as potential HDAC/VEGFR inhibitors. The first method involved the alkylation of the tetracyclic derivatives **46**, **47** obtained by the interaction of substituted anthranilamides and 2-formylbenzoic acids, with ethyl 6-bromohexanoic acid (Scheme 17). An attempt to obtain tetracyclic quinazolines **48** from NH-derivatives **46a**,**b** was unsuccessful. In the case of hydroxy derivatives **47a**,**b**, the alkylation in the presence of cesium carbonate in DMF resulted in the selective formation of products **49a**,**b** in good yields.

**Scheme 18:**
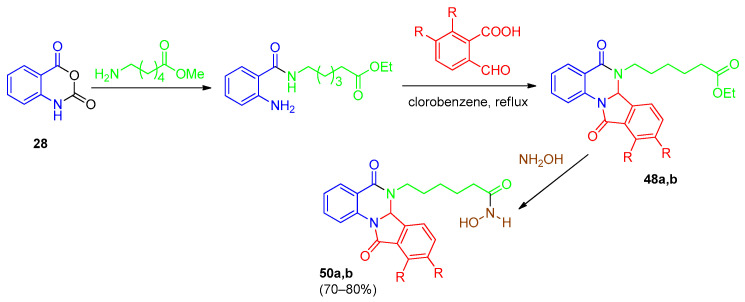
Synthetic approach to dihydroisoindolo[2,1-*a*]quinazolin-5,11-diones **50a**,**b**. **48**, **50**: R = H (**a**), OMe (**b**).

Another approach was selected for the synthesis of dihydroisoindolo[2,1-*a*]quinazolin-5,11-diones **48a**,**b** (Scheme 18). The reaction between isatoic anhydride **28** and ethyl 6-aminohexanoic acid led to substituted anthranilamide, which further converted into *N*-substituted tetracycles **48a**,**b** under the action of 2-formylbenzoic acids. The reaction of compounds **48a**,**b** with hydroxylamine enabled the synthesis of the expected derivatives **50a**,**b**, wherein hydroxamic acids were linked to tetracyclic quinazolinone-containing fragments via a spacer able to occupy the enzyme channel.

**Scheme 19:**
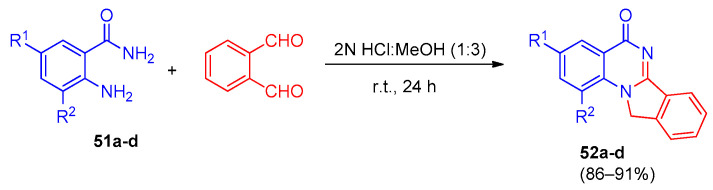
Synthesis of isoindolo[2,1-*a*]quinazolin-5(11*H*)-ones **52a**–**d** from anthranilamides **51a**–**d** (in blue) and *o*-phthalaldehyde (in red). **51**, **52**: R^1^, R^2^ = H, H (**a**); 3-NO_2_, H (**b**); 3-Br, H (**c**); Br, Br (**d**).

Mondal et al. described one-pot synthesis of isoindole-fused quinazolin-4-ones in the presence of an acid catalyst [45]. Interaction of substituted anthranilamides **51a**–**d** with *o*-phthalaldehyde in methanol and 2 N HCl in 3:1 ratio at room temperature led to the formation of range of isoindolo[2,1-*a*]quinazolin-5(11*H*)-ones **52a**–**d** (Scheme 19). It should be noted that compound **52a** was isolated from the reaction mixture as the salt **52a**·HCl, and **52a** was obtained only after the treatment with sodium bicarbonate solution. The structure of **52a**·HCl was confirmed by X-ray data; the tetracyclic skeleton is planar, and the hydrogen bonding of the amide hydrogen through a water molecule forms a network in the solid. Derivatives **52b**–**d** were isolated from the reaction as major products.

Moreover, Mondal et al. [45] performed the mechanistic study under deuterated solvent and revealed that the reaction proceeds through intramolecular 1,3-hydride shift. In addition to benzamides **51a**–**d**, various other amides such as 2-aminobenzenesulfonamide and 2-aminotetrahydrobenzothiophenamide were involved in this reaction, leading to corresponding analogs of compounds **52** with similarly high yields. Importantly, the type of substituents present in the amide group has little effect on the yield of the final products, thereby providing significant latitude for varying the structure of the bicyclic core. Additionally, this method could potentially be applied to many different variations of *o*-phthaldialdehyde compounds.

## 4. Pyrazolo[1,5-*a*]quinazolines and Indazolo[2,3-*a*]quinazolines

In the review article [14], some data on the synthetic approaches and bioactivity of pyrazoloquinazolines in which the pyrazole ring is attached to different edges of pyrimidine core are discussed. Regarding the synthesis of pyrazolo[1,5-*a*]quinazolines, two strategies were reported: the interaction of 2-hydrazinylbenzoic acid with 3-oxoalkanenitrile in the presence of CH_3_COOH [46] and the three-component reaction of 3(5)-amino-4-phenylpyrazole with aromatic aldehydes and cyclohexanone in acetic acid [47]. Some pyrazolo[1,5-*a*]quinazolines were described as negative allosteric modulators of metabotropic glutamate receptors [48], other tricyclic derivatives exhibit the topoisomerase-1 inhibitory activity [49]. Example synthesis of indazoloquinazoline system with *a*, *b*, or *c* patterns of annelation of indazole fragment to quinazoline core are provided in the micro-review [50].

**Scheme 20:**
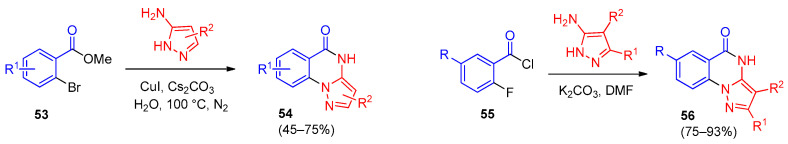
Synthetic approach to pyrazolo[1,5-*a*]quinazolines **54** and **56**. **54**: R^1^ = H, Cl, OMe, NO_2_; R^2^ = H, Me, CN, Ph, cyclopropyl, 2-thienyl; **56**: R = H, NO_2_; R^1^ = H, Me, COOEt, cyclopropyl, 4-MeC_6_H_4_, 2-thienyl; R^2^ = H, CN.

A series of new manuscripts on pyrazolo[1,5-*a*] and indazolo[2,3-*a*]quinazolines have been published more recently. Zhang et al. [51] presented a convenient and simple synthetic procedure for obtaining several pyrazolo[1,5-*a*]quinazolin-5(4*H*)-ones **54** via copper-catalyzed cascade reactions of 2-bromobenzoates **53** with 1*H*-pyrazol-5-amines under ligand-free conditions in water (Scheme 20). This method is based on commercially available starting materials; water is used as solvent, so the procedure responds to the urgent need for “greener” and “cleaner” chemistry.

Gnanasekaran et al. [52] incorporated 2-fluoroaroyl chlorides **55** into reaction with 5-aminopyrazoles (Scheme 20). The formation of pyrazolo[1,5-*a*]quinazolin-5(4*H*)-ones **56** is described as two-step sequence: initial acylation of the C5 amino group of the pyrazole was performed in DMF at −10 °C and further heating to 140 °C, then S_N_Ar ring closure between N1 of the pyrazole and the 2-fluoroarylamide was performed. Products **56** were obtained in high yields regardless of the nature of substituents.

**Scheme 21:**
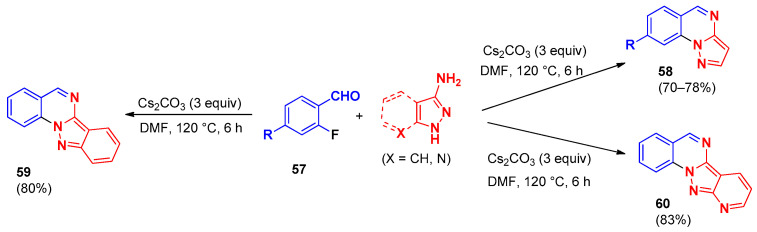
Synthesis of pyrazolo[1,5-*a*]quinazolines **58**, indazolo[2,3-*a*]quinazoline **59**, 10-azaindazolo[2,3-*a*]quinazoline **60**. **57**, **58**: R = H, Br, CF_3_.

An efficient method for the synthesis of pyrazolo[1,5-*a*]quinazolines **58**, indazolo[2,3-*a*]quinazoline **59**, and 10-azaindazolo[2,3-*a*]quinazoline **60** was described using various 2-fluorobenzaldehydes **57** interacting with 1*H*-pyrazol-3-amines or 1*H*-indazol-3-amine (and its 7-aza derivative) under metal-free conditions (Scheme 21) [53]. The process involves an intermolecular condensation step followed by a metal-free base-promoted intramolecular C–N coupling reaction. This methodology opens wide opportunities for preparation of various polycyclic quinazoline derivatives. Significantly, the bromine atom remains unaffected throughout the reaction process, allowing its subsequent utilization for additional chemical modifications or transformations.

**Scheme 22:**
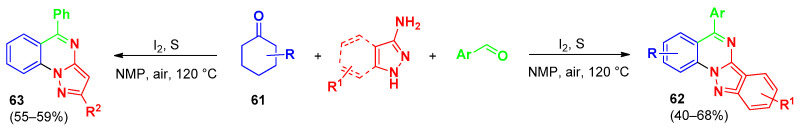
Synthesis of indazolo[2,3-*a*]- and pyrazolo[1,5-*a*]quinazolines **62**, **63**. **62**: R = H, F, OH, Cl, Br, Me, CN; R^1^ = H, Me, Et, *n*-Pr, CF_3_, OMe, COOEt, Ph, cyclohexyl-4-Pr; Ar = Ph, substituted Ar, naphthalene-2-yl, antracen-9-yl, hetaryl; **63**: R^2^ = H, CONH_2_, CN, COOEt.

Gao et al. [54] developed regioselective synthesis of indazolo[2,3-*a*]quinazolines **62** via a sequential annulation and dehydrogenative aromatization of cyclohexanones **61** (Scheme 22). The reaction is carried out in 1-methyl-pyrrolidin-2-one (NMP) as a solvent in the presence of iodine and sulfur at 120 °C for 20 h. A control experiment verified the sequential nature of the process steps. This reaction demonstrates broad compatibility across various types of aminoindazole compounds, encompassing those derived from aromatic and heterocyclic aldehydes, along with diversely functionalized cyclohexanones. Furthermore, this methodology has been successfully employed for the preparation of pyrazolo[1,5-*a*]quinazoline derivatives **63**.

**Figure 3:**
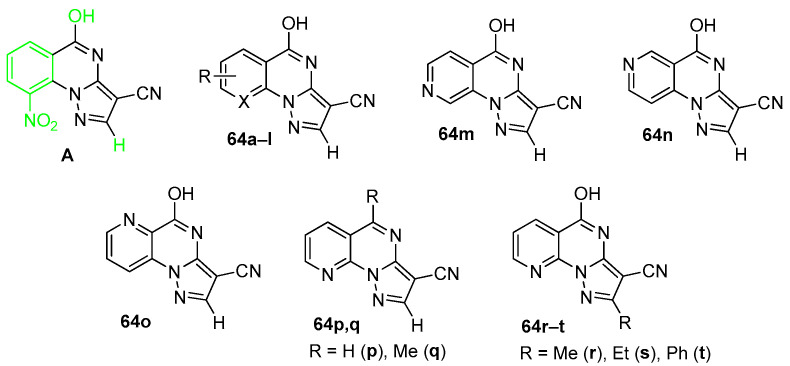
Chemical structures of compounds **A** and **64a**–**t**. **64a**–**l**: X = N, R = 8-Cl (**a**), 7-NO_2_ (**b**), 8-CF_3_ (**c**), H (**d**), 8-Me (**e**); X = CH, R = H (**f**), 8-MeSO_2_ (**g**), 8-CF_3_ (**h**), 9-CF_3_ (**i**), 8-NO_2_ (**j**), 9-NH_2_ (**k**), 8-NH_2_ (**l**).

A series of pyrazolo[1,5-*a*]quinazolines and their aza-analogues were synthesized and studied as inhibitors of histone lysine demethylase 4D (KDM4D) [55]. Initially, Fang et al. performed molecular docking for 30 compounds, two tricyclic derivatives showed an inhibition rate greater than 50% against KDM4D at a concentration of 10 µM. One of the leading compounds was pyrazolo[1,5-*a*]quinazoline derivative (**A**); its activity was measured towards three additional KDM enzymes (KDM2B, KDM3B, and KDM5A), and excellent selectivity toward KDM4D was demonstrated. Structural modifications focusing on three fragments of compound **A** (highlighted in green color) led to the synthesis of a series of derivatives **64a**–**t**. These derivatives were prepared by reacting 2-chloroacetyl chlorides, derived from either benzoic acid or picolinic acid, with corresponding 3-amino-*1H*-pyrazole-4-carbonitriles (Figure 3).

**Figure 4:**
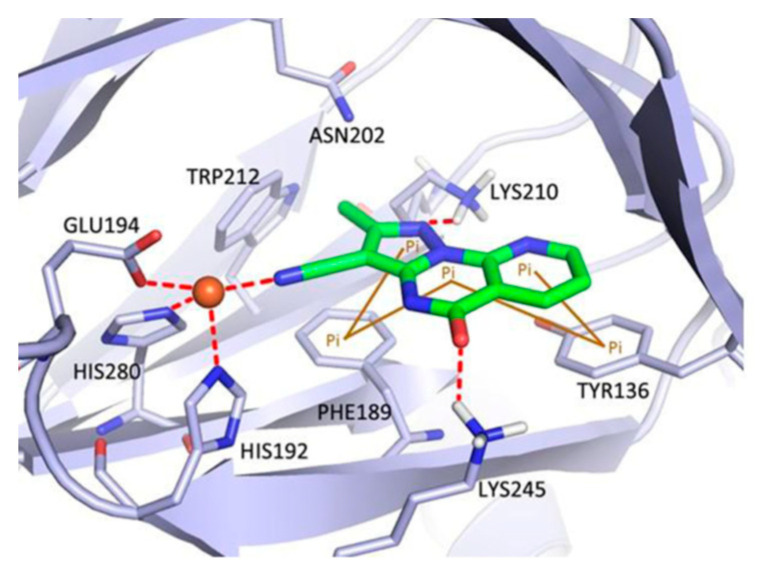
3D docking model of compound **64r** in active pocket of KDM4D. Compound **64r** is color coded with carbon atom by green, nitrogen atom by blue, and oxygen atom by red; Fe(II) is in orange and residues of KDM4D are in light blue. Reproduced with permission of Elsevier [55].

All target compounds were initially tested for their inhibitory activity against KDM4D at a concentration of 10 μM. Only three compounds (**64p**, **64r**, and **64s**) exhibited higher potencies compared to **A**. For these three compounds, IC_50_ values were determined, derivative **64r** was chosen as lead compound (Table 1). Moreover, quinazoline **64r** exhibits good selectivity for KDM4D; molecular docking was used to predict the binding model of compound **64r** in the active pocket of KDM4D (Figure 4).

**Scheme 23:**
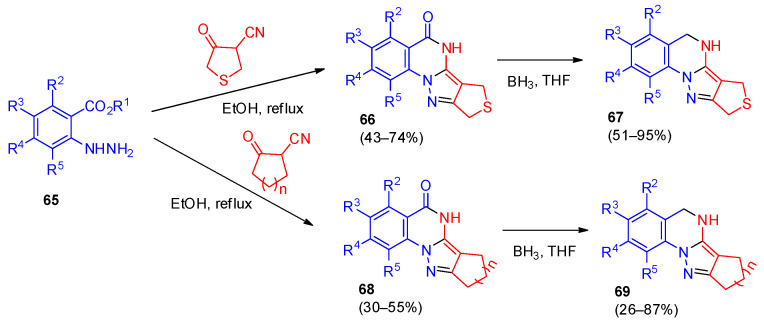
Synthesis of pyrazolo[1,5-*a*]quinazoline derivatives **66**–**69**. **65**–**69**: R^1^ = H, Me; R^2^ = H, Me, Br; R^3^ = H, Me, OMe; R^4^ = H, Me; R^5^ = H, Me; **68**, **69**: n = 1, 2.

Kovács et al. [56] reported the synthesis of pyrazolo[1,5-*a*]quinazoline derivatives through the reaction of 2-hydrazinobenzenecarboxylic acids or their esters (compounds **65**) with α-oxo-cyanides (Scheme 23). Refluxing a mixture of compound **65** and 4-oxotetrahydrothiophene-3-carbonitrile in ethanol led to the formation of 6,7-dihydro-*5H,9H*-thieno[3′,4′:3,4]pyrazolo[1,5-*a*]quinazolin-5-ones **66**, which were subsequently converted into amines **67**.

Interaction of 2-oxocyclopentanecarbonitrile (n = 1) or 2-oxocyclohexanecarbonitrile (n = 2) with arylhydrazines **65** in ethanol afforded a series of novel tetracyclic pyrazolo[1,5-*a*]quinazoline derivatives **68**, the reduction of the latter leads to formation of amines **69**. Notably, when R^2^ = Me, Br or R^5^ = Me, the yield of pyrazolo[1,5-*a*]quinazoline derivatives **66** and **68** significantly decreased, and their corresponding amines **69** were not detected.

**Scheme 24:**
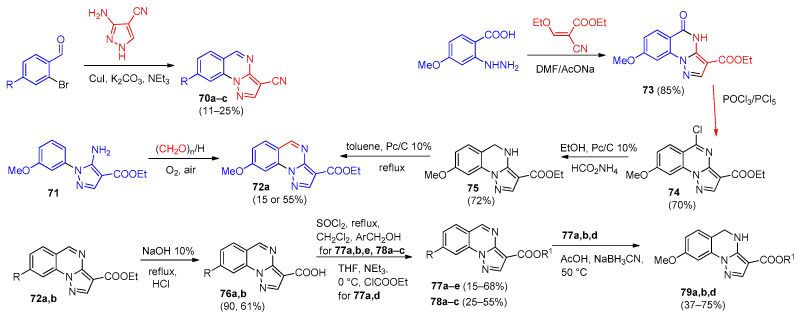
Synthetic approach to pyrazolo[1,5-*a*]quinazolines **70**, **72**, **73**–**75**, **76**, **77**, **78**, **79**. **70**: R = OMe (**a**), H (**b**), O(CH_2_)_2_OMe (**c**); **72**: R = OMe (**a**), H (**b**); **76**: R = OMe (**a**), H (**b**); **77**: R = OMe, R^1^ = CH_2_Ph (**a**), CH_2_-(2-OMe)Ph (**b**), CH_2_-2-thienyl (**c**), CH_2_-2-furyl (**d**), CHMe_2_ (**e**); **78**: R = H, R^1^ = CH_2_Ph (**a**), CH_2_-(2-OMe)Ph (**b**), CH_2_-2-thienyl (**c**); **79**: R^1^ = CH_2_Ph (**a**), CH_2_-(2-OMe)Ph (**b**), CH_2_-2-furyl (**d**).

5-Substituted pyrazolo[1,5-*a*]quinazolines were synthesized and evaluated as ligands of GABA_A_ receptor [57], only one compound showed receptor recognition in the nanomolar range. To find derivatives with higher activity, novel 3- and/or 8-substituted pyrazolo[1,5-*a*]quinazolines were synthesized [58]. Using a copper-catalyzed tandem reaction, appropriate 2-bromo-4-R-benzaldehyde and 5-amino-*1H*-pyrazole-4-carbonitrile, 3-cyanoderivatives **70a**–**c** were isolated in low yields (Scheme 24). Later [59], derivative **70b** was obtained in 55% yield by fusion of 5-amino-*1H*-pyrazole-4-carbonitrile with 2-chlorobenzaldehyde at 160 °C for 6 h. Interaction of ethyl 1-(3-methoxyphenyl)-5-aminopyrazole-3-carboxylate **71** with formaldehyde or paraformaldehyde in an acidic medium afforded 8-methoxy-pyrazolo[1,5-*a*]quinazoline **72a**; however, separation it from its 6-methoxy isomer was difficult. The key intermediate **72a** was synthesized in higher yield from 2-carboxy-5-methoxyphenylhydrazine via intermediate 8-methoxy-pyrazolo[1,5-*a*]quinazolines **73**, **74**, and **75** (Scheme 24). Derivative **72a** and ethyl pyrazolo[1,5-*a*]quinazoline-3-carboxylate **72b** were hydrolyzed to give the respective 3-carboxylic acids **76a**,**b**, which were further transformed into 3-ester derivatives **77a**–**e** and **78a**–**c**. Finally, compounds **77a**,**b**,**d** were converted into the corresponding 4,5-dihydroderivatives **79a**,**b**,**d**.

The in vitro study of the BZ site/GABA_A_-R binding affinity of synthesized pyrazolo[1,5-*a*]quinazolines derivatives **72**, **77**, **78** showed that the new compounds have binding recognition in the nanomolar range (3.16 < Ki (nM) < 529.3) at fixed concentrations of 10 μM, raising the subnanomolar affinity value of 0.27 nM for compound **77b** (Table 2). The pharmacological tests evidenced for compound **77b** a profile of positive allosteric modulator with anxiolytic and antihyperalgesic activity, lacking toxicity when tested in human neuronal-like cells and in vivo models.

**Scheme 25:**
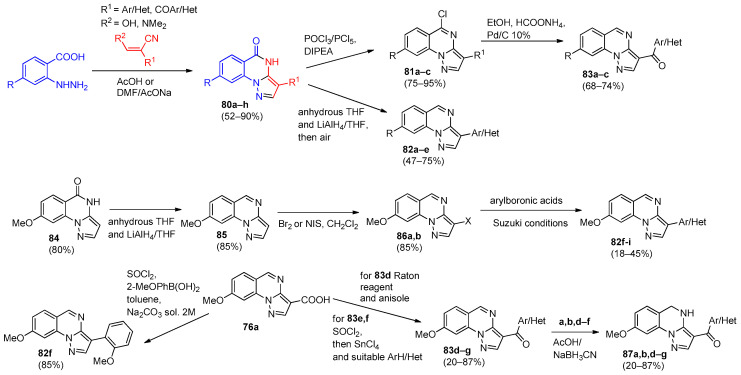
Synthetic approach to pyrazolo[1,5-*a*]quinazolines **80**, **82**, **83**, **87**. **80**: R = H, R^1^ = Ph (**a**), 2-thienyl (**b**), 3-thienyl (**c**); R = OMe, R^1^ = 2-thienyl (**d**), 3-thienyl (**e**), CO-(2-OMe)-Ph (**f**), 2-CO-2-thienyl (**g**), CO-3-thienyl (**h**); **82**: R = H, Ar/Het = Ph (**a**), 2-thienyl (**b**), 3-thienyl (**c**); R = OMe, Ar/Het = 2-thienyl (**d**), 3-thienyl (**e**), 2-MeOPh (**f**), 3-furyl (**g**), 1-Boc-2-pyrrolyl (**h**), *1H*-2-pyrrolyl (**i**); **83**: R = OMe, Ar/Het = (2-MeO)Ph (**a**), 2-thienyl (**b**), 3-thienyl (**c**), (4-MeO)Ph (**d**), 2-furyl (**e**), 2(*1H*)-pyrrolyl (**f**), 2-(1-methyl)pyrrolyl (**g**); **86**: X = Br (**a**), I (**b**); **87**: Ar/Het = 2-MeOPh (**a**), 2-thienyl (**b**), 4-MeOPh (**d**), 2-furyl (**e**), 2(*1H*)-pyrrolyl (**f**), 2-(1-methyl)pyrrolyl (**g**).

In the next work of the same team [60], modification of pyrazolo[1,5-*a*]quinazolines **77** was performed by shortening or removing the linker between aryl(hetaryl) ring and the pyrazolo[1,5-*a*]quinazoline core, 3-aryl(hetaryl)-pyrazolo[1,5-*a*]quinazolines **82** and 3-(hetero)aroyl-pyrazolo[1,5-*a*]quinazolines **83** were developed. Cyclization of hydrazinobenzoic acid with appropriate propanenitrile resulted in the formation of pyrazolo[1,5-*a*]quinazolines bearing at position 3 (hetero)aryl group (compounds **80a**–**e**) or (hetero)aroyl group (compounds **80f**–**h**) (Scheme 25). Treatment of compounds **80a**–**e** with LiAlH_4_ in anhydrous THF followed by oxidation led to 3-(hetero)aryl derivatives **82a**–**e**. Compounds **80f**–**h** were transformed into 3-(hetero)aroylpyrazolo[1,5-*a*]quinazolines **83a**–**c** via intermediate chloroquinazolines **81**.

The synthesis of compounds **82f**–**i** was accomplished using the Suzuki cross-coupling based on halogenated derivatives **86a**,**b**, which were obtained from 8-methoxypyrazolo[1,5-*a*]quinazoline **85** (Scheme 25). The starting quinazolinone **84** was synthesized by decarboxylation of derivative **73**. Derivative **76a** used for the preparation of quinazolines **82f** and **83d**–**g** (Scheme 25). Treating 3-(hetero)aroyl-pyrazolo[1,5-*a*]quinazolines **83a**,**b**,**d**–**g** with sodium borohydride complex (NaBH_3_CN) in AcOH allowed obtaining their dihydro analogs **87a**,**b**,**d**–**g**.

All target compounds were previously evaluated for their ability to displace [*3H*]flumazenil (Ro-151788) from its specific binding to Bz receptors in bovine membrane samples. Electrophysiological studies on recombinant α1ꞵ2γ2L GABA_A_ receptors were carried out for more promising compounds **82d**–**f**, **83a**–**d**, **86f**,**g**, **87a**,**b**,**d**. Quinazolines **82** bearing Ar/Het group could not modulate the GABA_A_ function, but they were found to act as null modulators or antagonists. Among aroyl/Het derivatives **83a** and **83b** can modulate the GABA_A_ receptor in an opposite manner: **83b** enhances and **83a** reduces the variation of the chlorine current. The most potent derivative **87d** reached a maximal activity at 1 μM (+54%) and enhanced the chlorine current at ≥0.01 μM. Moreover, compound **83g** demonstrated the ability to antagonize the full agonist diazepam.

**Scheme 26:**
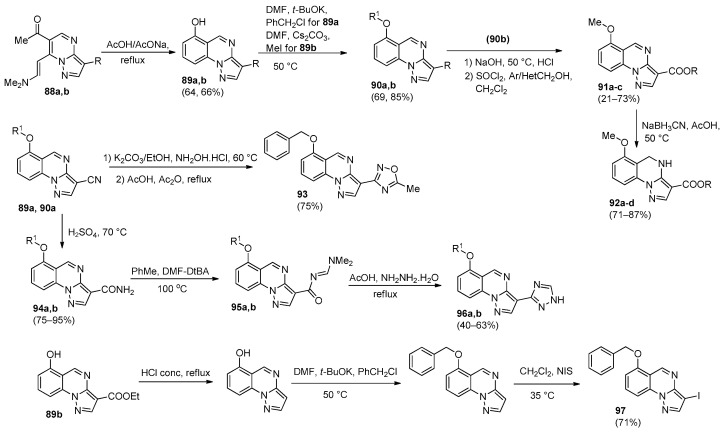
Synthetic approach to pyrazolo[1,5-*a*]quinazolines **89**–**94**, **96**, **97**. **89**: R = CN (**a**), COOEt (**b**); **90**: R, R^1^ = CN, CH_2_Ph (**a**), COOEt, Me (**b**); **91**: R = CH_2_Ph (**a**), CH_2_(2-OMe-Ph) (**b**), CH_2_-2-furyl (**c**); **92**: R = CH_2_Ph (**a**), CH_2_(2-OMe-Ph) (**b**), CH_2_-2-furyl (**c**), Et (**d**); **94**: R = H (**a**), CH_2_Ph (**b**); **96**: R = H (**a**), CH_2_Ph (**b**).

Guerrini et al. [61] evaluated the effect of the shift of the methoxy group from position 8 to position 6, with the same (hetero)aryl ester groups at position 3. 6-Acetyl-7-(2-dimethylaminovinyl)pyrazolo[1,5-*a*]pyrimidine 3-cyano or 3-ethoxycarbonyl **88a**,**b**, obtained according to the described method [62], were used for the synthesis of 3,6-disubstituted pyrazolo[1,5-*a*]quinazolines. Boiling of these compounds in an acetic buffer solution led to the formation of 6-hydroxypyrazolo[1,5-*a*]quinazolines **89a**,**b**, whose alkylation yielded derivatives **90a**,**b** (Scheme 26). Compound **90b** was hydrolyzed to produce the corresponding 3-carboxylic acid, which was esterified to ethers **91a**–**c**, and then reduced to 4,5-dihydroderivatives **92a**–**d**. Compound **90a** under the treating with hydroxylamine hydrochloride in ethanol and potassium carbonate, followed by cyclization was transformed into 3-(5-methyl-1,2,4-oxadiazol-3-yl)-derivative **93**. To prepare derivatives **96a**,**b**, compounds **89a** and **90a** were converted into 3-carboxyamide derivatives **94a**,**b**, which upon reaction with dimethylformamide-di-*tert*-butylacetate (DMF-DtBA) in toluene formed acylamidines **95a**,**b**. Boiling these acylamidines with hydrazine hydrate yielded compounds **96a**,**b**. 3-Iodo-6-benzyloxypyrazolo[1,5-*a*]quinazoline **97** was obtained from compound **89b** after decarboxylation, alkylation, and iodination (Scheme 26).

Novel 3,6-disubstituted pyrazolo[1,5-*a*]quinazolines **89**–**94**, **96**, **97** were studied as ligands to GABA_A_ receptor. The GABA_A_-binding affinity of compounds was evaluated for their ability to displace [^3^H]flumazenil (Ro-151788) from its specific binding in a bovine membrane. From the obtained results, it follows that the compounds demonstrated the percent of inhibition of specific [3H]Ro15-1788 binding at 10 μM concentration from 1% to 43.7%, iodine-containing compound **97** showed the highest inhibition (43.7%), quinazoline **89b** demonstrated 32% inhibition (Table 3). Drawing upon both experimental evidence and computational molecular modeling analyses conducted on compound **91b**, the researchers inferred that relocating substituent groups from position 8 to position 6 plays a critical role in achieving effective ligand-receptor interactions.

**Figure 5:**
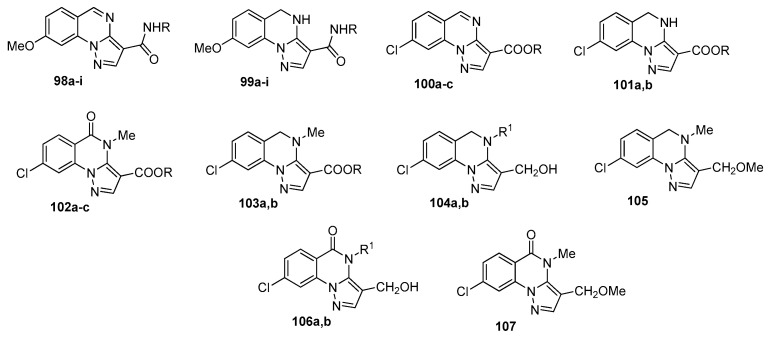
Chemical structures of compounds **98**, **99**, **100**–**107**. **98**, **99**: R = H (**a**), CHMe_2_ (**b**), cyclohexyl (**c**), CH_2_cyclohexyl (**d**), Ph (**e**), CH_2_Ph (**f**), CH_2_(2-OMe)Ph (**g**), CH_2_(2-furyl) (**h**), CH_2_(2-thienyl) (**i**); **100**: R = CH_2_(2-OMe)Ph (**a**), CH_2_(2-thienyl) (**b**), t-Bu (**c**); **101**: R = CH_2_(2-OMe)Ph (**a**), CH_2_(2-thienyl) (**b**), Et (**c**); **102**: R = CH_2_(2-OMe)Ph (**a**), CH_2_(2-thienyl) (**b**), *t*-Bu (**c**); **103**: R = CH_2_(2-OMe)Ph (**a**), CH_2_(2-thienyl) (**b**), Et (c); **104**: R^1^ = H (**a**), Me (**b**); **106**: R^1^ = H (**a**), Me (**b**).

New 8-methoxypyrazolo[1,5-*a*]quinazolines **98a**–**i** and their dihydro derivatives **99a**–**i** bearing the amide fragment at the position 3 were synthesized [63] as analogues of 8-methoxypyrazolo[1,5-*a*]quinazoline 3-ester [57,58], 3-(hetero)aroyl and 3-(hetero)aryl derivatives [60] identified as modulators of GABA_A_ receptors. 8-Methoxypyrazolo[1,5-*a*]quinazoline-3-carboxylic acid **78a** after the treatment with thionyl chloride or trichloroacetonitrile/PPh_3_ in CCl_4_ led to the formation of Het-C(O)Cl intermediate, which without isolation interacted with the appropriate amine, giving compounds **98a**–**i**, which were transformed into the corresponding 4,5-dihydroderivatives **99a**–**i** by the treatment with sodium cyanoborohydride (NaBH_3_CN) in acetic acid (Figure 5).

All the new compounds **98a**–**i** and **99a**–**i** have been evaluated in vitro for their ability to modulate the chlorine current on recombinant GABA_A_ receptors of the α1β2γ2L type (expressed in frog oocytes of the *Xenopus laevis* species). Two groups of compounds were identified from electrophysiological test: positive modulators agonists (**98e**,**h**,**i** and **99e**,**h**) and null modulators antagonist (**98a**,**b**,**d**,**f**,**g** and **99a**–**d**,**f**,**g**).

In the next work [64], new 8-chloropyrazolo[1,5-*a*]quinazoline derivatives **100**–**107** (Figure 5) were presented as GABA_A_ receptor modulators. Their syntheses resemble those illustrated in Scheme 24 and Scheme 25. Compounds **100**–**107** underwent molecular dynamics simulations performed on an isolated segment of the GABA_A_ receptor protein located between α and γ chains, where the benzodiazepine-binding site is identified. Using the ‘Proximity Frequencies’ model (PF), Crocetti et al. [63] predicted that compounds **100a**, **103a**, and **106b** belong to the agonist class with 93.1% probability. On the contrary, derivatives **101c**, **103c**, and **107** fall into the antagonist class, with 62–73% prediction. Thus, two types of compounds occupy different areas in the binding site. The virtual prediction for **106b** and **107** as agonist and antagonist, respectively, was confirmed through electrophysiological assays.

**Figure 6:**
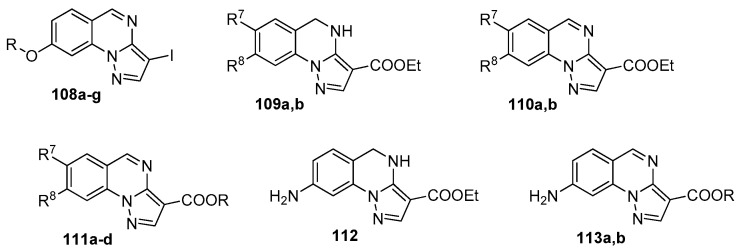
Chemical structures of compounds **108**–**113**. **108**: R = Me (**a**), CH_2_*c*Pr (**b**), propyne (**c**), CH_2_Ph (**d**), CH_2_(2-MePh) (**e**), CH_2_(2-MeOPh) (**f**), CH_2_(pyridin-4-yl) (**g**); **109**: R^7^ = H, R^8^ = NO_2_ (**a**), R^7^ = NO_2_, R^8^ = H (**b**); **110**: R^7^ = H, R^8^ = NO_2_ (**a**), R^7^ = NO_2_, R^8^ = H (b); **111**: R^7^ = H, R^8^ = NO_2_, R = CH_2_(2-MeOPh) (**a**), R^7^ = H, R^8^ = NO_2_, R = CH_2_-thien-2-yl (**b**), R^7^ = NO_2_, R^8^ = H, R = CH_2_(2-MeOPh) (**c**), R^7^ = NO_2_, R^8^ = H, R = CH_2_-thien-2-yl (**d**); **113**: R = CH_2_(2-MeOPh) (**a**), CH_2_-thien-2-yl (**b**).

3,8-Disubstituted pyrazolo[1,5-*a*]quinazoline **108**–**113** (Figure 6), bearing oxygen or nitrogen function at position 8, were synthesized and studied as GABA_A_ receptor modulators [65]. The synthesis is based on approaches presented in Scheme 24 and Scheme 25.

**Figure 7:**
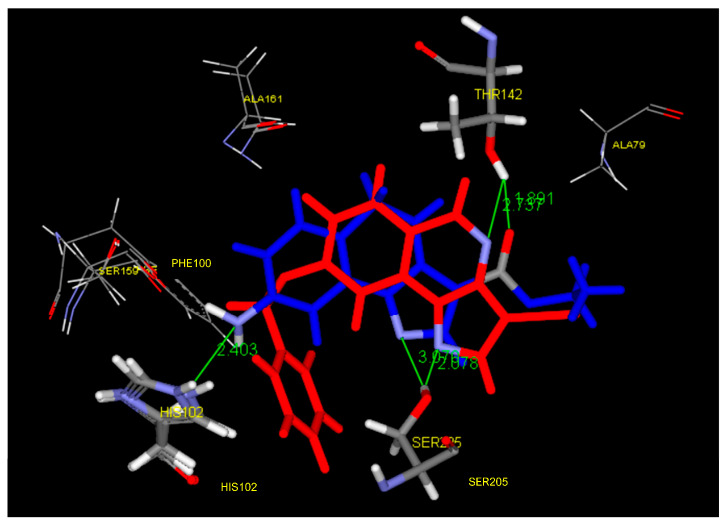
Hydrogen bonds among agonist **112** (blue), antagonist **108d** (red), and γThr142, αHis102, and αSer205 in the binding site. Reproduced with permission of MDPI [65].

Compounds **108**–**113** were screened through electrophysiological techniques on recombinant α1β2γ2L-GABA_A_ receptors expressed in *Xenopus laevis* oocytes, some compounds exhibited certain ability to bind the receptor. The most promising electrophysiological results were obtained for compounds **108d**, **109a**, **109b**, and **112**. Among the 3-iododerivatives, compound **108d**, which does not modulate the chlorine current, was evaluated for its ability to antagonize the full agonist lorazepam (1 μM). Compounds **109a** and **112** were found to exhibit agonist profile while quinazolines **109b** and **108d** act as antagonists. Molecular modelling studies and Hierarchical Cluster Analysis (HCA) data have collocated these ligands in the class corresponding to their pharmacological profile (Figure 7).

**Scheme 27:**
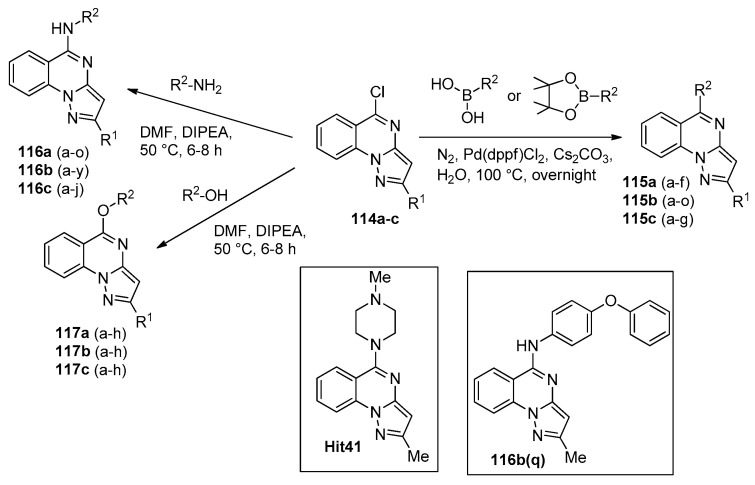
Synthesis of pyrazolo[1,5-*a*]quinazoline derivatives **115a** (a-f), **115b** (a-o). **115c** (a-g); **116a** (a-o), **116b** (a-y), **116c** (a-j); **117a** (a-h), **117b** (a-h), **117ca** (a-h). R^1^ = H (**a**), Me (**b**), *t*Bu (**c**); R^2^ = Ar, Ar-Ar, Ar-O-Ar, thiazolyl, benzothiazolyl, benzooxazolyl, pyrazinyl etc.

A large series (102 compounds) of pyrazolo[1,5-*a*]quinazoline derivatives were designed and synthesized to investigate their activity as SIRT6 activators [66]. SIRT6 is a particularly important member of Sirtuins and has emerged as a novel therapeutic target for various diseases. Previously [67], a 2-methyl-5-(4-methylpiperazin-1-yl)pyrazolo[1,5-*a*]quinazoline **(Hit41)** had been found as a new SIRT6 de-fatty-acylation activator. Zhang et al. [66] discovered highly active SIRT6 de-fatty-acylation activators by structural modifications of Hit41, focusing on expanding substituents at positions 2 (R^1^) and 5 (R^2^) of the pyrazolo[1,5-*a*]quinazoline scaffold. Three series of derivatives were synthesized (Scheme 27): **115a**–**117a** (R^1^ = H); **115b**–**117b** (R^1^ = Me); **115c**–**117c** (R^1^ = *t*Bu). In each series, R^2^ represented a broad set of Ar or Het substituents (number indicated in parentheses). Chlorinated precursors **114a**–**c**, obtained using one of the approaches shown in Scheme 24, were used as starting materials.

Fluor de Lys (FDL) assays were performed for compounds **115**–**117**, for some compounds additional calculations were made to determine the concentration of the compound (μM) at which the compound can increase the enzymatic activity by 50% values (EC_1.5_), structure–activity relationship (SAR) was studied. A set of novel SIRT6 activators was obtained (13 compounds with EC_1.5_ < 50.79 μM); among them, 2-methyl-*N*-(4-phenoxyphenyl)pyrazolo[1,5-*a*]quinazolin-5-amine **116b(q)** is the most potent one, which exhibited excellent defatty-acylation activation activity against SIRT6 (EC_1.5_ 1.85 ± 0.41 μM and EC_50_ 11.15 ± 0.33 μM). Notably, that the calculated EC_1.5_ value for Hit41 is only 49.30 ± 0.74 μM. The bioactivity of **116b(q)** was further verified by differential scanning fluorimetry (DSF) and surface plasmon resonance (SPR) assays. Molecular docking showed that the pyrazolo[1,5-*a*]quinazoline **116b(q)** formed a hydrogen bond with Val115 and four π–π interactions with Phe64, Phe82, and Phe86; **116b(q)** can significantly improve the thermal stability of SIRT6 protein and inhibit the PI3K/Akt signaling pathway in mouse embryonic fibroblasts (MEFs), thereby inhibiting the proliferation of MEFs. As a result, Zhang et al. [66] discovered a new potent SIRT6 activator, which can be taken as a lead compound for later studies.

**Scheme 28:**
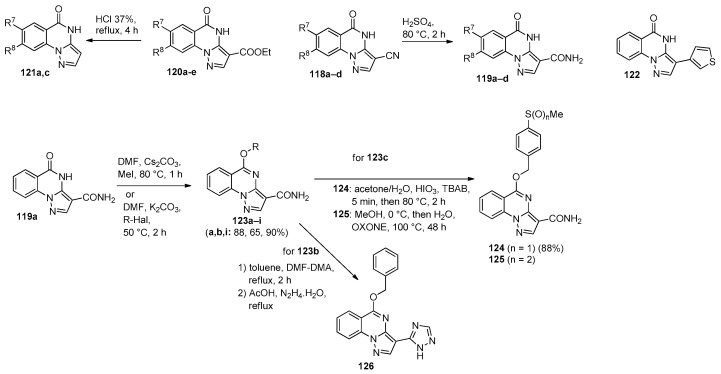
Synthesis of pyrazolo[1,5-*a*]quinazoline derivatives **118a**–**d**, **119a**–**d**, **120a**–**e**, **121a**–**c**, **122**, **123a**–**e**, **124**, **125**, **126**. **118**–**121**: R^7^, R^8^ = H, H (**a**); H, Cl (**b**), H, NO_2_ (**c**); NO_2_, H (**d**); H, OMe (**e**); **123**: R = Me (**a**), CH_2_-Ph (**b**), CH_2_-4-SMe-Ph (**c**), CH_2_-2-Cl-Ph (**d**), CH_2_-3-Cl-Ph (**e**), CH_2_-4-Br-Ph (**f**), CH_2_-2-Me-Ph (**g**), CH_2_-3-OMe-Ph (**h**), CH_2_-4-SO_2_NH_2_-Ph (**i**).

A library of new pyrazolo[1,5-*a*]quinazoline compounds substituted at positions 3, 7, or 8 was synthesized and their anti-inflammatory activity was studied [68]. The key intermediates **118a**–**d**, **120a**–**e**, and **122** were obtained by the condensation of disubstituted 2-hydrazinobenzoic acid with ethoxymethylene-malononitrile, ethyl-2-cyano-3-ethoxyacrylate, and 3-oxo-2-(3-thienyl)proponitrile, respectively (Scheme 28). Acid hydrolysis of nitriles **118** yielded amides **119**, while carboethoxy derivatives **120** were converted into unsubstituted counterparts **121**. Pyrazolo[1,5-*a*]quinazolines **118**–**122** were subjected to further transformations, and 45 compounds were synthesized for screening. For example, from derivative **119a**, a series of 5-alkoxy-substituted pyrazolo[1,5-*a*]quinazolines **123a**–**i** was prepared, the compound **123c** was oxidized to yield sulfoxide **124** and then sulfone **125**, whereas **123b** was transformed into 3-(1,2,4-triazole) derivative **126** (Scheme 28).

Compounds **123**–**126** were tested for their ability to inhibit lipopolysaccharide (LPS)-induced nuclear factor κB (NF-κB) transcriptional activity in human THP-1Blue monocytic cells. Only 13 compounds were able to inhibit NF-κB activity with IC_50_ < 50 μM, two of them showed the highest activity: **123i** (5-[(4-sulfamoylbenzyl)oxy]pyrazolo[1,5-*a*]quinazoline-3-carboxamide) and **124** (5-[(4-(methylsulfinyl)benzyloxy]pyrazolo[1,5-*a*]quinazoline-3-carboxamide). Pharmacophore mapping of potential targets and molecular modeling allowed to conclude that these compounds could effectively bind to extracellular signal-regulated kinase 2 (ERK2), p38α, and c-Jun N-terminal kinase 3 (JNK3) with the highest complementarity to JNK3. Moreover, compounds **123i** and **124** exhibited micromolar binding affinities for JNK1, JNK2, and JNK3. Obtained results [68] demonstrate the potential for developing lead anti-inflammatory drugs of pyrazolo[1,5-*a*]quinazoline nature.

**Scheme 29:**
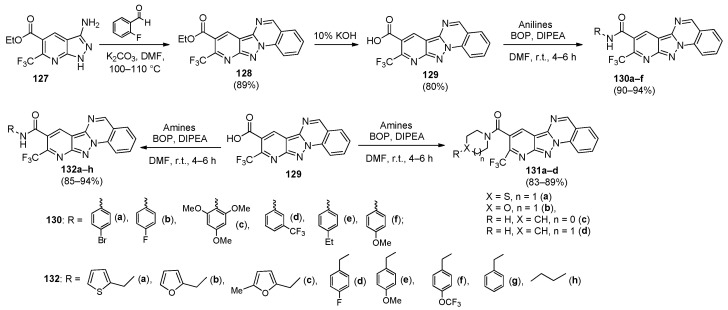
Synthetic approach to 9-(trifluoromethyl)-pyrido[2′,3′:3,4]pyrazolo[1,5-*a*]quinazoline derivatives **128**–**132**.

Kumar et al. presented the synthesis of tetracyclic hybrid structures **127**–**132**, combining pyrazolo[3,4-*b*]pyridine and pyrazolo[1,5-*a*]quinazoline fragments, and data of their biological activity [69]. Reaction of 3-amino-pyrazolo[3,4-*b*]pyridine-5-carboxylate **127** with 2-fluorobenzaldehyde resulted in the formation of ethyl pyrido[2′,3′:3,4]pyrazolo[1,5-*a*]quinazoline-8-carboxylate **128** in 89% yield. Hydrolysis of compound **128** under alkaline conditions produced carboxylic acid derivative **129**, which upon reaction with various substituted anilines, cyclic secondary amines, and primary aliphatic amines led to the corresponding amide-substituted pyridopyrazolo-quinazolines **130a**–**f**, **131a**–**d**, and **132a**–**h** (Scheme 29). In all cases, the reaction between **129** and amines was carried out in the presence of benzotriazol-1-yloxy-tris(dimethylamino)phosphonium hexafluorophosphate (BOP).

Pyridopyrazolo-quinazolines **128**–**132** were evaluated for antibacterial activity against Gram-positive and Gram-negative bacterial strains, compounds **129**, **130a**, **130c**, **130f**, **131a**, **132c**, **132f**, and **132h** exhibited promising antibacterial activity against various bacterial strains (Table 4). Compound **129** showed high antibacterial (MIC 3.9 μg/mL) and broad-spectrum anti-biofilm activity. Compounds **129** and **132b** also showed good antifungal activity against various *Candida* strains. Compound **129** was found to be promising as a broad-spectrum biofilm inhibitor both against bacterial pathogens and against *C. albicans* MTCC3017. Further, compound **129** reduced the ergosterol content in three *Candida* strains (*C. albicans* MTCC227, *C. albicans* MTCC1637, and *C. albicans* MTCC3017). Both compound **129** and Miconazole showed the same amount of inhibition of ergosterol content in *C. albicans* MTCC227, modelling studies were also performed for validation. Compounds **131d**, **132a**, **132b**, and **132d** exhibited inhibition > 90% against MCF7 (breast) cancer cell line; compounds **130d**, **131a**, **132a**, **132b**, **132f**, and **132g** exhibited inhibition > 90% against SKOV3 (ovarian) cancer cell line, Doxorubicin was used as a standard.

**Scheme 30:**
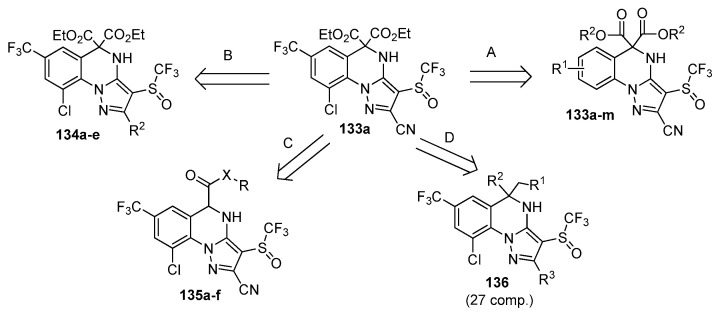
Synthetic approach to 4,5-dihydropyrazolo[1,5-*a*]quinazolines **133**–**136**. **133**: R^1^ = CF_3_, NO_2_, CN, Cl, Br, F, OCF_3_; R^2^ = Et, Me; **134**: R^2^ = Me, Et, *n*-Pr, cyclobutylmethyl, 4-chlorobenzyl; **135**: X = O, NH; R = Me, Et, CH_2_CF_3_, 2,4-Me_2_Ph, 2-Cl-5-Me-thiazole, 2-Cl-5-Me-Py; **136**: R^1^ = esters, amides, acylsulfur; R^2^ = H, esters; R^3^ = SOCF_3_ or SO_2_CF_3_.

Synthesis and insecticidal activities of polysubstituted 4,5-dihydropyrazolo[1,5-*a*]quinazolines **133**–**136** (Scheme 30) were described [70,71,72]. 5,5-Disubstituted 4,5-dihydropyrazolo[1,5-*a*]quinazoline derivative **133a**, obtained by interaction of 5-amino-1*H*-phenylpyrazole with dialkyl bromomalonate, exhibited promising insecticidal activity against *P. xylostella* [70]. Later, the same team [71] performed some modifications of compound **133a**: substituents in benzene ring (direction A), position 3 of the pyrazole ring (direction B), position 5 of the quinazoline ring (direction C), synthesized three series of novel 4,5-dihydropyrazolo[1,5-*a*]quinazoline derivatives (**133**–**135**). Afterwords, an expanded series of analogues **136** was obtained [72].

Insecticidal activities of compounds **133**–**135** against insect pests *P. xylostella*, *S. frugiperda*, and *S. invicta* were evaluated. The most compounds exhibited good (>50% mortality) to excellent (100% mortality) insecticidal effect against *P. xylostella*; fifteen compounds from the series showed significant mortality rate > 50% at 100 mg/L, and nine compounds were able to show mortality rate > 50% at 25 mg/L, with LC_50_ values found to be 3.87–24.35 mg/L. Compound **133** (R^1^ = CF_3_, Cl, R^2^ = Me) exhibited the best insecticidal activity; its LC_50_ value against *P. xylostella* (3.87 mg/L) was comparable to that of **indoxacarb** (4.82 mg/L), which is one of the major commercial pesticides to control *P. xylostella.* Insecticidal activities of compounds **133a** and **135** (X = NH, 2-Cl-5-Me-thiazole) against *S. frugiperda* (mortality rate 79.63% and 72.12%) were comparable to that of **fipronil** (mortality rate 68.44%). The compounds **133** (R^1^ = CF_3_, Cl, R^2^ = Me) and **135** (X = O, R = Me) showed high insecticidal activities against *S. invicta* (mortality rate 96.67% and 95.56%) comparable to that of **fipronil** (mortality rate 100%) after 5 days of treatment at 1.0 mg/L. Moreover, electrophysiological studies indicated that compound **136** (R^1^ = CO_2_Me, R^2^ = H, R^3^ = CN) could act as a potent GABA receptor antagonist (2 μΜ, inhibition 68.25%).

## 5. Imidazo[1,2-*a*]quinazolines, Benzimidazo[1,2-*a*]quinazolines

Synthetic approaches to imidazoquinazolines, in which imidazole cycle is attached to c, b or g edges of quinazoline core, as well as to benzo[4,5]imidazo[1,2-*c*]- and benzo[4,5]imidazo[1,2-*a*]quinazolines are described in book chapter [13]. Two main approaches to [*a*]-annelated derivatives are used: condensation of 2-aminobenzimidazole with various aromatic aldehydes bearing Hal or NO_2_; and intramolecular C–N bond formation. This section of the current manuscript contains data on the synthesis and biological activity of imidazo- and benzimidazo[*a*]quinazolines, which were not included in the book chapter [13] or appeared later.

**Scheme 31:**
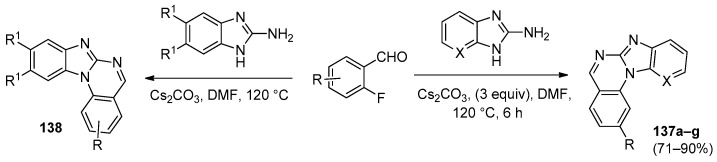
Synthetic approach to benzimidazo[1,2-*a*]quinazolines **137**, **138**. **137**: X = CH, R = H (**a**), Br (**b**), OMe (**c**), CF_3_ (**d**), NO_2_ (**e**); X = N, R = H (**f**), Br (**g**); **138**: R = F, Cl, Br, CF_3_, Me, OMe; R^1^ = H, Cl, Me.

Annareddygari et al. [53] developed an efficient method for the synthesis of benzo[4,5]imidazo[1,2-*a*]quinazolines **137** from different 2-fluoro-benzaldehydes with 2-aminobenzimidazole (its 7-aza derivative) under metal-free conditions in high yields (Scheme 31). The process includes an intermolecular condensation followed by metal-free base-promoted intramolecular C–N coupling reaction.

Using this approach, a series of trisubstituted benzo[4,5]imidazo[1,2-*a*]quinazolines **138** was obtained to study their anti-fungal activities against six plant pathogenic fungi and analyze SAR [73]. It was found that compound **138** (R = R^1^ = H) demonstrated broad-spectrum antifungal activities with EC_50_ = 4.43 μg/mL.

**Scheme 32:**
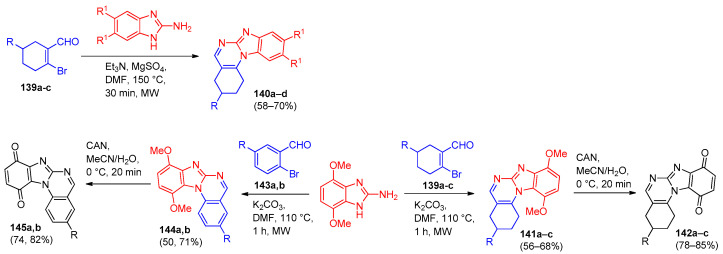
Synthetic approach to tetrahydrobenzo[4,5]imidazo[1,2-*a*]quinazolines **140**, **141**, benzimidazo[1,2-*a*]quinazolines **144**, and benzimidazole-4,7-dione derivatives **142a**–**c**, **145**. **140**: R, R^1^ = H, H (**a**), H, Me (**b**), Me, H (**c**), Ph, H (**d**); **141**, **142**: R = H (**a**), Me (**b**), Ph (**c**); **143**–**145**: R = H (**a**), F (**b**).

Microwave-assisted synthesis of 1,2,3,4-tetrahydrobenzo[4,5]imidazo[1,2-*a*]quinazolines **140** was presented **[74]**. The reaction of 2-bromocyclohex-1-ene carbaldehydes (**139a**–**c**) and 2-aminobenzimidazoles in the presence of both base and magnesium sulfate under microwave irradiation led to the formation of derivatives **140** in 58–70% yields (Scheme 32).

In the next work [75], 2-bromocyclohex-1-enecarbaldehydes **139a**–**c** were incorporated into the reaction with 4,7-dimethoxy-*1H*-benzimidazole-2-amine under optimized conditions (microwave irradiation) for the obtaining tetrahydrobenzo[4,5]imidazo[1,2-*a*]quinazolines **141** and subsequent oxidation (Scheme 32). It was demonstrated that 4,7-dimethoxy-1*H*-benzimidazole-2-amine also interacts effectively with 2-bromobenzaldehydes **143** with the formation of benzimidazo[1,2-*a*]quinazolines **144**. Oxidation of quinazoline-fused dimethoxybenzimidazoles **141** and **144** was conducted using ceric ammonium nitrate (CAN) in the MeCN/H_2_O medium. The corresponding benzo[4,5]imidazo[1,2-*a*]quinazoline-8,11-diones **142** and **145** were obtained in good yields.

**Scheme 33:**
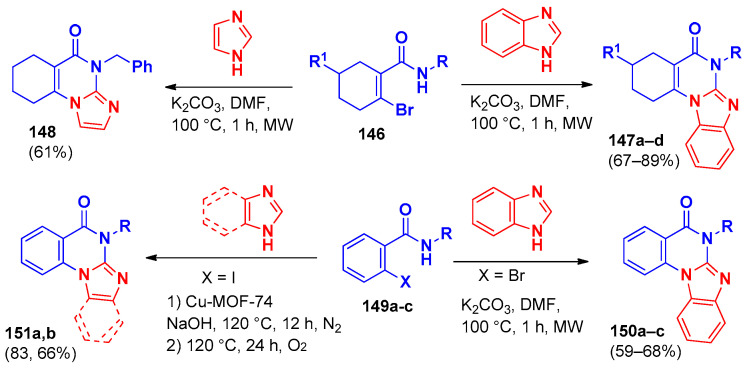
Synthetic approach to tetrahydrobenzo[4,5]imidazo[1,2-*a*]quinazolines **147**, tetrahydroimidazo[1,2-*a*]quinazolin-5-one **148**, benzimidazo[1,2-*a*]quinazolin-5-ones **150a**–**c**, **151a**, and imidazo[1,2-*a*]quinazolin-5-one **151b**. **147**: R, R^1^ = Bz, H (**a**); Bu, H (**b**); Bz, Me (**c**); Bz, Ph (**d**); **150**: R = Bz (**a**), Bu (**b**), Ph (**c**); **151a**: R = *n*-Pr, benzimidazole; **151b**: R = *n*-Bu, imidazole.

Dao et al. [76] reported the synthesis of 1,2,3,4-tetrahydrobenzo[4,5]imidazo[1,2-*a*]quinazolin-5-ones **147** from ꞵ-bromo-α,ꞵ-unsaturated amides and benzimidazole via copper powder catalyzed C–N coupling and subsequent C–N bond formative cyclization by C–H activation under microwave irradiation conditions (Scheme 33). Interaction of amide **146** with imidazole under the same conditions led to the formation of 1,2,3,4-tetrahydroimidazo[1,2-*a*]quinazolin-5-one **148**. It was also demonstrated that benzamides **149** under similar conditions allowed to obtain benzimidazo[1,2-*a*]quinazolin-5-ones **150a**–**c** (Scheme 33). This reaction provides the first known way to make these unique hybrid nitrogen-containing structures using common starting materials.

Heterogeneous amorphous Cu–MOF-74 catalyst was developed for C–N coupling reaction and applied for the synthesis of benzimidazo/imidazo[1,2-*a*]quinazolin-5-ones **151a**,**b** [77] (Scheme 33). Ma et al. demonstrated that crystalline Cu–MOF-74 can be used as a catalyst precursor to synthesize aCu-MOF-74 under the action of alkali and high temperature in the reaction solution, and then it acts as a true heterogeneous catalyst to catalyze C–N coupling between 2-iodobenzamides **149** and benzimidazole/imidazole, which gives higher isolated yields. The catalyst can be simply removed after the reaction and reused up to six times without losing much effectiveness.

**Scheme 34:**
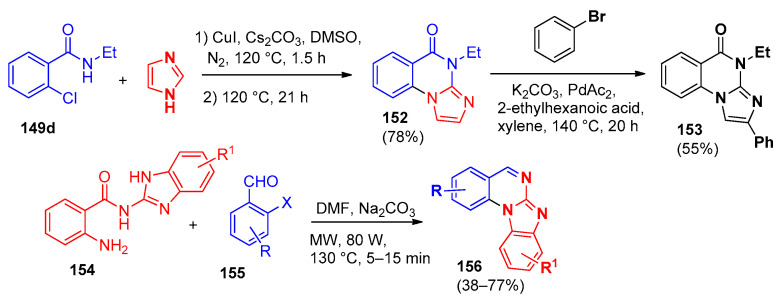
Synthesis of imidazo[1,2-*a*]quinazolin-5-one **153** and benzimidazo[1,2-*a*]quinazolines **156**. **154**: R^1^ = H, 5,6-Cl; **155**: X = F, Cl, Br, NO_2_; R = H, 4-F, 5-F, 4-Cl, 3-OMe, 5-Br, 4-Br, 3,6-Br.

An approach based on the Ullman coupling of 2-chlorobenzamide **149d** with imidazole followed by oxidative ring formation was used for the synthesis of imidazo[1,2-*a*]quinazolin-5-one **152** [78] (Scheme 34). When 4-phenylimidazole was applied, the yield of compound **153** was only 23%, while functionalization of derivative **152** by palladium-catalyzed direct C–H arylation with bromobenzene led to the formation of 2-phenyl-4-ethyl-imidazo[1,2-*a*]quinazolin-5-one **153** in 55% yield.

Rapid and efficient microwave-assisted metal-free base-mediated synthetic approach to the series of benzimidazo[1,2-*a*]quinazolines **156** (15 compounds) (Scheme 34) from readily available building blocks was developed [13,79]. In the presence of groups X = NO_2_ or R = 5-Br in the aldehyde **155**, the yields of compounds **156** decreased to 38% and 44%, respectively. This method uses easy-to-get starting materials, works with many different substrates, is simple to carry out, and allows making various benzimidazo[1,2-*a*]quinazoline compounds without needing metals.

**Scheme 35:**
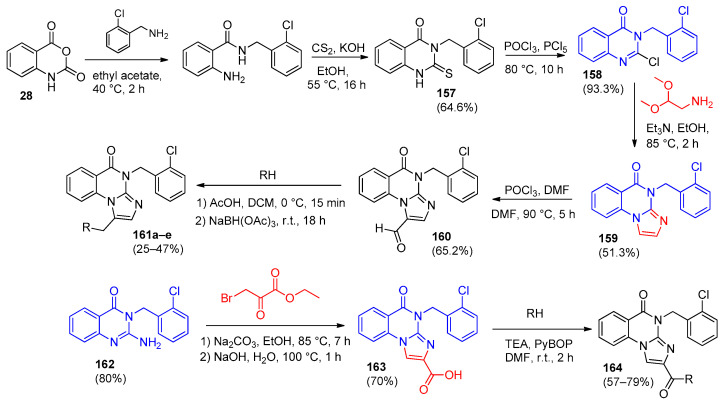
Synthetic approach to 4-(2-chlorobenzyl)imidazole[1,2-*a*]quinazolin-5(4*H*)-ones **159**, **161a**–**e**, **164**. **161**: R = 4-Me-piperazino (**a**), pyrrolidinyl (**b**), piperidinyl (**c**), morpholino (**d**), thiomorpholino (**e**); **164**: R = cycloimines, cycloamines, arylamines.

One more strategy for the synthesis of imidazole[1,2-*a*]quinazolin-5(4*H*)-ones was described [80]. Interaction of isatoic anhydride **28** with *o*-chlorobenzyl amine gave 2-amino-*N*-(2-chlorobenzyl)benzamide, which under the reaction with CS_2_ in the alkali medium was converted into 3-(2-chlorobenzyl)-2-thioxo-2,3-dihydroquinazolin-4(1*H*)-one **157** (Scheme 35). Heating this compound with a mixture of POCl_3_ and PCl_5_ yielded the corresponding 2-chloro derivative **158**, which upon reaction with 2,2-dimethoxyethylamine in the presence of NEt_3_ afforded 4-(2-chlorobenzyl)imidazo[1,2-*a*]quinazolin-5(*4H*)-one **159**. The structure of compound **159** was confirmed by various spectroscopic methods including X-ray crystallography.

In the next work [81] 4-(2-chlorobenzyl)imidazolo[1,2-*a*]quinazolin-5(4*H*)-one **159** was functionalized at imidazole cycle by the Vilsmeier–Haack reaction with DMF and POCl_3_ and then reductive amination with a secondary amine reagent to obtain compounds **161a**–**e** (Scheme 35). Second series of 4-(2-chlorobenzyl)imidazole[1,2-*a*]quinazolin-5(4*H*)-ones (**164**, 27 compounds) was synthesized based on 2-amino-3-(2-chlorobenzyl)quinazolin-4(3*H*)-one **162**, obtained from chloro derivative **158** in the reaction with *p*-methoxy-benzylamine. Removing the benzyl group, cyclizing **162** with ethyl bromopyruvate and condensing with amine compounds led to the formation of compounds **164** in good yields (Scheme 35).

**Figure 8:**
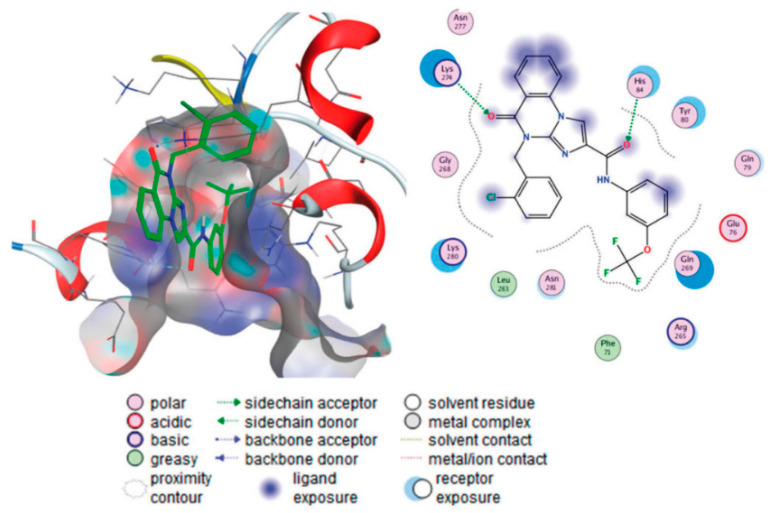
Interaction of compound **164** (R = 3-(OCF_3_)C_6_H_4_NH) with SHP2 protein. Reproduced with permission of Taylor & Francis [81].

Imidazoquinazolinone derivatives **161** and **164** were studied as allosteric inhibitors of SHP2 phosphatase, in vitro enzymatic assays were conducted. The inhibitory activities of **161** and **164** against SHP2 protease at 100 μM and 200 μM were evaluated; results demonstrated that most of the compounds have certain inhibitory activity against SHP2 protease. Imidazoquinazolinones **161a** (22.14% inhibition) and **164** (R = 4-HO-piperidinyl) (29.81% inhibition) showed better inhibition of SHP2 protein activity than SHP244 as a positive control, (14.95% inhibition) at 100 μM. The in vitro cytotoxicity of 100 μM concentrations of compounds **161**, **164** towards the melanoma cell line A375 was evaluated; according to results, compared with SHP244 and sorafenib (inhibition 13.81% and 14.79%), most of the compounds showed significant cytotoxicity to A375. Among them, **161a** (76.15% inhibition) and **164** (R = *m*-CF_3_-aniline) (27.93% inhibition) showed effective activity (Table 5). The IC_50_ values were also measured for the series of compounds, and generally it was shown that, compared with SHP244, the tumor cell activity of **161** and **164** compounds is significantly better than enzyme activity. The docking studies revealed that C=O groups of imidazoquinazolinones form hydrogen bond interactions with Lys274 and His84, respectively (Figure 8).

**Scheme 36:**
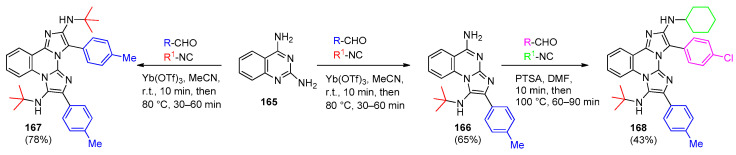
Synthesis of imidazo[1,2-*a*]quinazoline derivative **166**, diimidazo[1,2-*a*:1′,2′-*c*]quinazoline derivatives **167**, **168**.

Ghashghaei et al. [82] presented selective multiple multicomponent Groebke–Blackburn–Bienaymé reaction (GBBR), which allows to obtain imidazoazines by acid-catalyzed interaction of α-aminoazines, aldehydes and isocyanides. Thus, diaminoquinazoline **165** underwent a regioselective GBBR providing mono-adduct **166** at components ratio 1:1:1 (Scheme 36). In a different reaction, substrate **165** afforded the symmetrical bis-adduct **167** (components ratio 1:2:2). Moreover, a second GBBR, performed upon **166**, gave the non-symmetrical compound **168** (components ratio 1:1:1). This method can be widely applied and create complicated molecules selectively, adjustably, and directly.

**Scheme 37:**
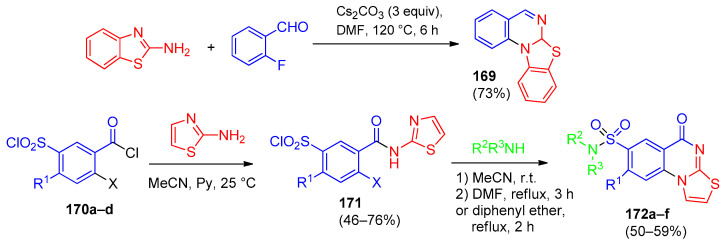
Synthesis of benzo[4,5]thiazolo[3,2-*a*]quinazoline **169**, thiazolo[3,2-*a*]quinazolin-5-ones **172**. **170**, **171**: X, R^1^ = F, H (**a**); Cl, H (**b**); Cl, Cl (**c**); Br, H (**d**); **172**: R^1^ = H (**a**–**c**), Cl (**d**–**f**); R^2^R^3^N = morpholin-4-yl (**a**,**d**); R^2^ = H, R^3^ = Ph (**b**,**e**); R^2^R^3^N = piperidin-4-yl (**c**,**f**).

Annareddygari et al. [53] developed the general approach to azoloquinazolines. Thus, the reaction of 2-aminobenzothiazole with 2-fluorobenzaldehyde led to the formation of benzo[4,5]thiazolo[3,2-*a*]quinazoline (**169**) in 73% yield under the optimized conditions (Scheme 37).

The series of thiazolo[3,2-*a*]quinazolin-5-one derivatives **172a**–**f** containing a sulfonamide group on C7 was described [83]. Interaction of chlorosulfonyl-substituted benzoyl halogenides **170a**–**d** with aminothiazoles led to the formation of amides **171** (Scheme 37). The treatment them with amine under mild conditions and subsequent cyclization under reflux in DMF or diphenyl ether allowed to obtain thiazolo[3,2-*a*]quinazolin-5-ones **172a**–**f**. The influence of substituent R^1^ and the nature of halogen on the yield of target products has been analyzed.

## 6. Triazolo[*a*]quinazolines

### 6.1. [1,2,4]-Triazolo[4,3-a]quinazolines

Book chapter [12] discloses approaches to triazolo[*a*]- and triazolo[*c*]-annelated quinazolines based on traditional transformations of chloroquinazolines, condensations between quinazoline and azide or nitrile, copper-catalyzed alkyne-azide cycloaddition reaction, multicomponent reactions, and microwave-assisted procedures. In the book chapter [12], some data on biological activity was also presented. The current section of the manuscript contains data on the synthesis and biological activity of triazolo[*a*]quinazolines, which were not included in the book chapter [12] or appeared later.

**Scheme 38:**
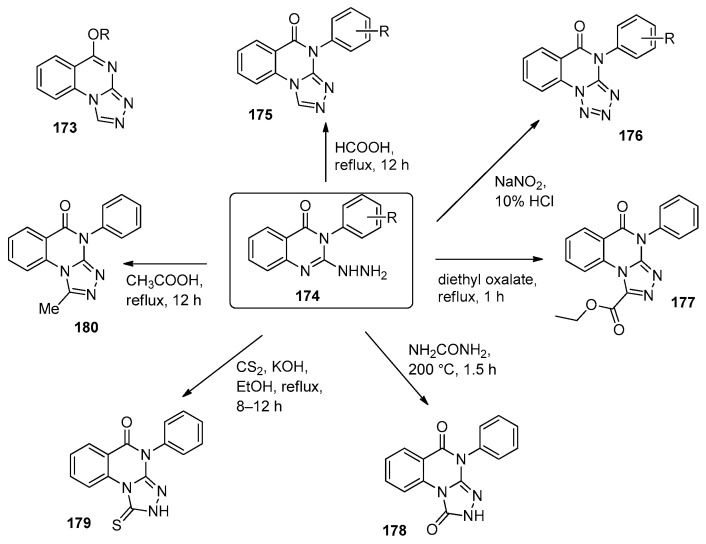
Synthesis of 4-aryl-[1,2,4]triazolo[4,3-*a*]quinazolin-5(*4H*)-ones **175** (24 compounds), **176** (23 compounds) and **177**–**180**. **174**–**176**: R = H, *o*(*p*)-OMe, *p*-OC_3_H_7_, *p*-OC_4_H_9_, *p*-OC_5_H_11_, *p*-OC_6_H_13_, *p*-OC_8_H_17_, *p*-OC_10_H_21_, *p*-OC_12_H_25_, *p*-OC_14_H_29_, *o*(*m*,*p*)-F, *o*(*m*,*p*)-Cl, *m*(*p*)-Br, *o*(*m*,*p*)-Me, *p*-CF_3_, *o*,*p*-Cl_2_.

A series of 5-substituted-[1,2,4]triazolo[4,3-*a*]quinazolines **173** was synthesized, and anticonvulsant activity was tested [84]. Among the compounds, **173** 5-heptyloxy-derivative was found to be especially potent (ED_50_ = 39.4 mg/kg, PI = 8.3). Continuing research in this direction, the same team has presented a new series of 4-aryl-[1,2,4]triazolo[4,3-*a*]quinazolin-5(*4H*)-ones **175** and **176**, 1-substituted derivatives **177**–**180** (Scheme 38), and evaluated their anticonvulsant [85] and antidepressant activities [86]. In these compounds, an aryl substituent is introduced into the NH group of quinazolin-4(*3H*)-one, unlike derivatives **173**, while the triazole ring is modified. 2-Hydrazino-3-phenyl-2,3-dihydroquinazolin-4(*1H*)-one derivatives **174**, obtained by slightly modifying a previously reported method [87], were used as starting materials for the synthesis of [1,2,4]triazolo[4,3-*a*]quinazolin-5(*4H*)-ones **175**–**180**. The treatment of derivatives **174** with formic acid under reflux overnight led to 4-aryl-[1,2,4]triazolo[4,3-*a*]quinazolin-5(4*H*)-ones **175** (24 compounds). The 4-aryl-[1,2,4]tetrazolo[4,3-*a*]quinazolin-5(*4H*)-ones **176** (23 compounds) were obtained by reaction **174** with NaNO_2_ in 10% HCl. To obtain the target compounds **177**–**180**, derivative **174** was reacted with diethyl oxalate, urea, carbon disulfide, and acetic acid, respectively (Scheme 38). The amido derivative **177** was obtained by treating ethoxycarbonyl derivative **176** with aqueous ammonia.

Compounds **175**–**180** were evaluated for their anticonvulsant activity and neurotoxicity by maximal electroshock (MES) and rotarod neurotoxicity tests [85]. The compounds were dissolved in DMSO and Kunming mice in the 18–22 g weight range were used. According to preliminary data, 11 compounds from the series of **175** possessed anticonvulsant activity against MES-induced seizures at a dose of 100 mg/kg, and five of these (R = *o*(*m*,*p*)-Cl, *p*-Br, 3,4-Me_2_) were found to remain active at a dose of 30 mg/kg. Further studies demonstrated that two derivatives **175** (R = *p*-Cl and *p*-Br) displayed wide spectrum activity in several models (Table 6), exhibited wide margin of safety with a protective index (PI) exceeding one for existing drugs (>25.5 and >26.0), and showed significant oral activity against MES-induced seizures in mice, with ED_50_ 88.02 and 94.60 mg/kg, respectively.

Compounds **175**–**180** were evaluated for antidepressant activities in mice [86]. Most of them showed antidepressant activity in the forced swimming test (FST). It was found that three compounds from the series of **176** (R = *o*-F, *m*-Me and *p*-Me) at a dose of 50 mg/kg significantly reduced the immobility time in the FST. Compound **176** (R = *p*-Me) proved to be the most active, the immobility time decreased by 82.69% at 50 mg/kg dose (Table 6). Also, this substance did not change normal behavior in tests, and its positive impact was like the antidepressant fluoxetine.

**Scheme 39:**
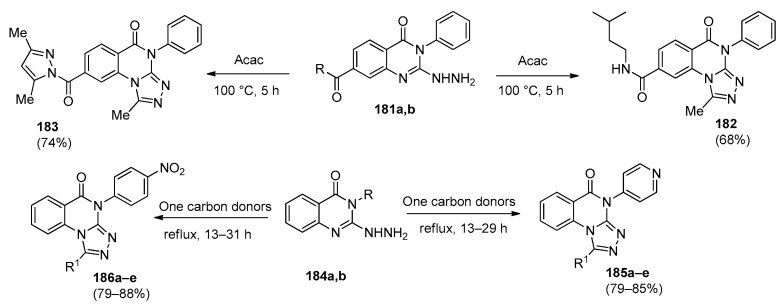
Synthesis of 1-substituted-[1,2,4]triazolo[4,3-*a*]quinazolin-5(4*H*)-ones **182**, **183**, **185a**–**e**, **186a**–**e**. **181**: R = NHNH_2_ (**a**), NH-*i*C_5_H_11_ (**b**); **184**: R = pyridine-4-yl (**a**), 4-nitrophenyl (**b**); **185**, **186**: R^1^ = H (**a**), Me (**b**), Et (**c**), *n*-Pr (**d**), CH_2_Cl (**e**).

1-Methyl[1,2,4]triazolo[4,3-*a*]quinazolin-5(4*H*)-ones **182**, **183** were obtained by the reaction of 2-hydrazinoquinazolin-4(3*H*)-ones **181a**,**b** with acetylacetone (Scheme 39) [90]. Notably, the 7-hydrazinocarbonyl group in quinazolin-4(3*H*)-one **181a** is transformed into a pyrazole derivative, which can be replaced by amine with the amide formation.

1-Substituted-[1,2,4]triazolo[4,3-*a*]quinazolin-5(4*H*)-ones, bearing at position 4 pyridine-4-yl (**185a**–**e**) or *p*-nitrophenyl (**186a**–**e**) fragment (Scheme 39) were described [91,92]. Compounds **185a**–**d** and **186a**–**d** were obtained by the reaction of 2-hydrazino-3-R-quinazolin-4(3*H*)-one **184** with the corresponding acid as one carbon donor, whereas interaction of **184** with chloroacetyl chloride in glacial acetic acid was used for the preparation of compounds **185e** and **186e**. Both series of derivatives have been evaluated for their in vivo antihistaminic and sedative–hypnotic activities. The protection against histamine-induced bronchospasm on conscious guinea pigs method was adopted to determine the antihistaminic potential of the test compounds. All compounds exhibit significant antihistaminic activity, the percentage protection for compounds **185a**–**e** is 69–73%, and for derivatives **186a**–**e** 68–71.6%. The highest activity was shown by derivatives **185a** and **186a**. Sedative-hypnotic activity was determined by measuring the reduction in motor activity. The results showed that almost all the test compounds were found to exhibit mild activity (less than 10%).

**Scheme 40:**
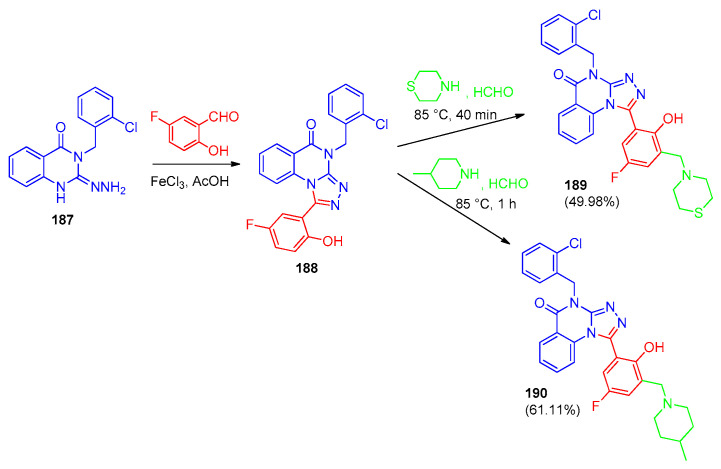
Synthesis of 4-(2-chlorobenzyl)containing [1,2,4]triazolo[4,3-*a*]quinazolin-5(4*H*)-ones **189** and **190**.

The synthesis, crystal structure, DFT study, and molecular docking of two new 4-(2-chlorobenzyl)-containing [1,2,4]triazolo[4,3-*a*]quinazolin-5(4*H*)-ones (**189**, **190**) were presented [93,94]. Both derivatives were synthesized according to Scheme 40 starting from the corresponding 4(*3H*)-quinazolinone-3-[(2-chlorophenyl)methyl]-2-hydrazonyl **187**, obtained via hydrazinolysis of quinazoline thione **157** (see Scheme 35). The structures of derivatives **189** and **190** have been confirmed by X-ray diffraction data. The crystal structures of **189**, **190** were compared with the conformers optimized by DFT calculation, and they were found to be consistent. Studies on the molecular electrostatic potential and frontier molecular orbital (FMO) of **189** show that this compound has a certain nucleophilic reactivity and large hardness.

Molecular docking study for compound **189** demonstrated the formation of seven hydrogen bonds with SHP2 protein, indicating that this compound has a better binding effect with SHP2 [93]. Molecular docking of **190** suggests favorable interactions with SHP2 protein [94]. The SHP2 enzyme inhibitory activity of **190** was evaluated at 10 μM, (**SHP244** was used as a reference compound [95]) and it was shown that activity (inhibition rate 12.40%) was lower than that of **SHP244** (19.67%). The antitumor activity of compound **190** was evaluated in human hepatoma cells SMMC7721, human melanoma cells A375 and human breast cancer cells MCF-7 using an MTT assay (**SHP244** was used as a reference compound). It was found that the IC_50_ values were 757, 70.19 and >1000 μM, respectively. Compound **190** has better selectivity for melanoma cells. The antitumor activity of **190** was better than that of the reference compound in SMMC7721 and A375, which may be due to **190** has better physical and chemical properties.

**Scheme 41:**
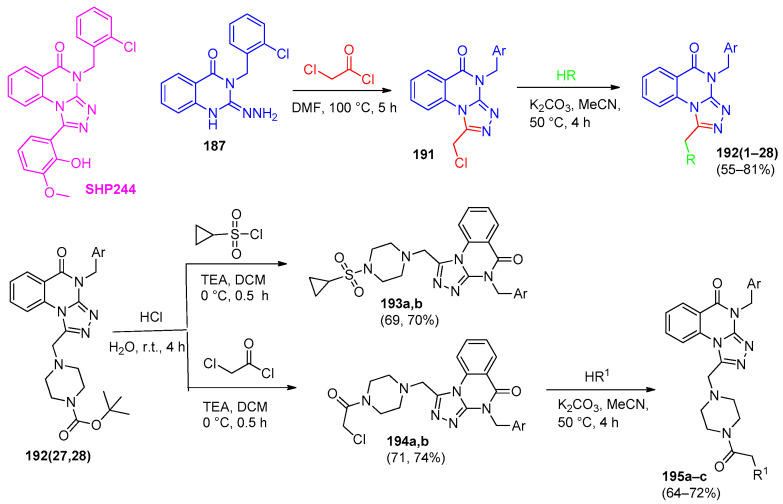
Synthesis of [1,2,4]triazolo[4,3-*a*]quinazolin-5(4*H*)-one derivatives **192**, **193**, **195**. **192**: Ar = (furan-2-yl)methyl (**1-6**), (thiophen-2-yl)methyl (**7**–**10**), 2-chlorobenzyl (**11**), 4-fluorobenzyl (**12**–**16**, **27**), 4-methoxybenzyl (**17**–**26**, **28**); **193**, **194**: Ar = 4-fluorobenzyl (**a**), 4-methoxybenzyl (**b**); **195**: Ar, R^1^ = 4-fluorobenzyl, 2-morpholinoacetyl (**a**), 4-methoxybenzyl, 2-morpholinoacetyl (**b**), 4-methoxybenzyl, 2-(pyrrolidin-1-yl)acetyl (**c**).

A series of [1,2,4]triazolo[4,3-*a*]quinazolin-5(*4H*)-one derivatives were synthesized and studied as SHP2 protein inhibitors [95]; among them, compound SHP244 showed good results. Continuing these studies, new groups of [1,2,4]triazolo[4,3-*a*]quinazolin-5(*4H*)-ones (compounds **192(1**–**28)**, **193a**,**b**, **195a**–**c**) were obtained through modification of the SHP244 structure at positions 1 and 4 (Scheme 41) [81]. The benzene ring in the triazole cycle was replaced with a nitrogen-containing side chain to enhance the binding effect with the receptor and the 2-chlorobenzene ring was replaced with an aromatic ring containing different substituents to investigate the effect of this part of the structure on the activity.

**Figure 9:**
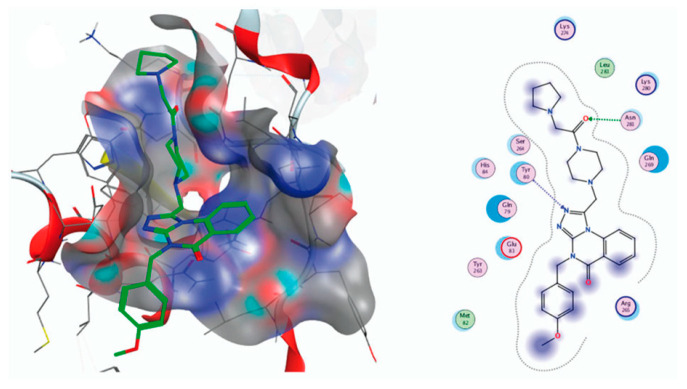
Interaction of compound **195c** with SHP2 protein. Compound **195c** was shown in colored sticks (green: carbon atom, blue: nitrogen atom, red: oxygen atom). Reproduced with permission of Taylor & Francis [81].

The inhibitory activities of triazoloquinolinone derivatives **192**, **193**, and **195** against SHP2 protease at 100 mM and 200 mM were evaluated in vitro. Self-isolated and purified SHP2 was used as the target protein, and SHP244 was used as a positive control. It was shown that most compounds have certain inhibitory activity against SHP2 protease. Compared with SHP244, some compounds show similar or higher sensitivity to SHP2, which indicates that the modification of the three parts of SHP244 has a profound influence on the activity. For example, compound **193b** (28.20% inhibition) and compound **195b** (26.38% inhibition) showed better SHP2 protein inhibitory activity than comparable structures at 100 mM, and better than SHP244 (23.52% inhibition). To study the antitumor activity of the compounds **192**, **193**, **195**, SHP244, and sorafenib were used as positive controls, and the MTT method was used to evaluate the in vitro cytotoxicity of the compounds 100 mM on the melanoma cell line A375 (Table 6). Compared with SHP244 and sorafenib, most of the compounds showed significant cytotoxicity to A375, such as **192(5)** (27.02% inhibition) and **192(21)** (29.60% inhibition). Metabolic stability of compound **192(22)** in human and rat liver microsome in vitro was studied, SHP244 was used as a positive control. It was found that this compound showed considerable stability in human and rat liver microsomes, and the clearance rate for both human and rat liver microsomes was less than 9.6 (mL/min/mg) with similar remaining (82.0% and 78.9%, t_1/4_ 60 min). At the same time, t_1/2_ are both greater than 145 min, which is much greater than t_1/2_ of SHP244 for human and rat liver microsomes (14.2 and 12.2 min). Docking studies of the compound **195c** was performed, and it was shown that C(O) group of 2-(pyrrolidin-1-yl)acetyl fragment and nitrogen of triazole cycle form hydrogen with the amino acid residues Asn281 and Tyr80, respectively (Figure 9).

**Figure 10:**
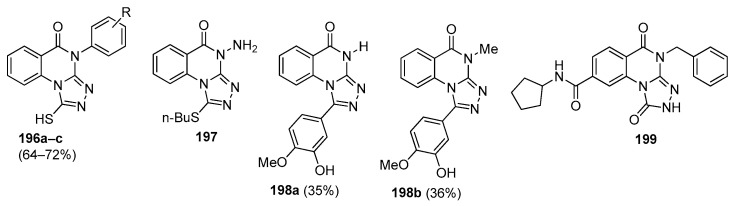
Chemical structures of compounds **196**, **197**, **198a**,**b**, and **199**. **196**: R = H (**a**), 4-OEt (**b**), 4-Me (**c**).

Some [1,2,4]triazolo[4,3-*a*]quinazolin-5(4*H*)-one derivatives exhibit other types of biological activity. Thus, compounds **196a**–**c** (Figure 10) were screened for their in vitro antimicrobial and antitubercular activities against pathogenic strain [96]. Compounds were obtained by the reaction of 3-aryl-2-hydrazinoquinazolines with carbon disulfide in good yields. Compound **196b** was found to be nearly equipotent with Ciprofloxacin against both Gram-positive and Gram-negative bacteria (*P. aeruginosa* and *S. aureus*) with MIC 2.6 μg/mL and demonstrated very promising antifungal activity against *A. niger* with MIC 2.6 μg/mL. The anti-tubercular activity of compounds **196** reveal that compound **196b** showed better activity than the other compounds with a MIC of 5.2 μg/mL against *M. tuberculosis* H_37_Rv.

A 3D similarity-based virtual screen to search for ligands of Toll-like receptor (TLR7), an important target for drug discovery, was performed [97]. Six new compounds were identified as interesting initial hit compounds that can act as TLR7 antagonists with micromolar potencies, as determined using a reporter assay. Among them, [1,2,4]triazolo[4,3-*a*]quinazolin-5(4*H*)-one **197** (Figure 10), possessing good solubility, was progressed into biological evaluation and showed antagonistic activity (IC_50_ = 185 μM).

A series of 1-aryl-[1,2,4]triazolo[4,3-*a*]quinazolin-5-ones **198** (11 derivatives) designed as conformationally restricted of CA-4 (well-known antimitotic agent) analogues was synthesized to study their biological efficiency toward tubulin polymerization, their cytotoxicity toward various cancer cell lines, and their antivascular effects [98]. Derivatives **198** were obtained by the reaction of 3-benzyl(methyl)-2-hydrazino-quinazolinones with corresponding aryl aldehydes followed by oxidative cyclization. Only two of them (**198a**,**b**) were potent in vitro tubulin polymerization inhibitors with IC_50_ values of 4.26 and 0.15 mM respectively, but only the *N*-methyl substituted counterpart **198b** displayed strong growth inhibitions toward the NCI-60 human cancer cell lines panel. This compound has shown remarkable activity in the HUVEC shape change assays as cell invasion and endothelial tube formation experiments which are good indicators for potential in vivo antivascular activity. These results suggest that compound **198b** might be lead compound for the development of novel vascular disrupting agents and is promising candidates for in vivo evaluation.

Triazolo[4,3-*a*]quinazolin-5-one derivative **199** demonstrated potent NF-κB inducing kinase (NIK) inhibitory effect with IC_50_ values of 103.4 ± 5.1 μM and 57% [99]. Identification of new NIK inhibitors by discriminatory analysis-based molecular docking was performed, 70 compounds from different classes were selected and tested by the NIK adenosine triphosphate (ATP) consumption essay for NIK inhibitory activities. Three of them, including derivative **199**, have an inhibiting rate over 50%.

**Figure 11:**
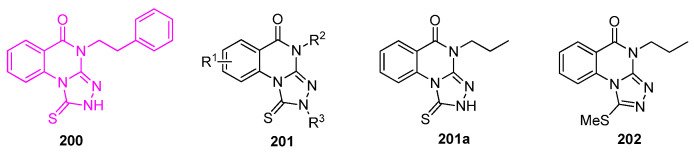
Chemical structures of compounds **200**, **201**, and **202**.

Identification of a new heterocyclic scaffold for inhibitors of the Polo-Box Domain of Polo-like Kinase 1 (PBD of Plk1) was performed [88]. A small chemical library of ∼400 drug-like molecules was screened for the ability to bind to the PBD of Plk1. Among four hits identified was triazoloquinazolinone **200** (Figure 11), which inhibited PBD binding in the ELISA assay with an IC_50_ of 4.38 μM. When compared to the previously characterized phosphopeptide, PLHSpT (IC_50_ of 14.74 μM), the affinity of the compound **200** is anticipated to be at least threefold higher than that of peptide. To find compounds with improved characteristics within this series, a large range of triazolo[4,3-*a*]quinazolin-5-ones **201** (147 derivatives) was synthesized by modifying different fragments of structure **200**. All derivatives were obtained through the reaction of 3-R-2-hydrazino-quinazolinones with carbon disulfide. Various substituents were introduced as R^1^ groups (7(8,9)-F, 7-Br, 7-I, 7-Me, 7-NHAc, 7-NMe_2_) and aza-analogues of the phenyl ring were also introduced. Many alkyl, heteroalkyl, and arylalkyl modifications of the R^2^ group (phenylethyl in compound **200**) were included.

All compounds were evaluated for their efficacy against the full-length human Plk1 in an ELISA assay and for their in vitro physiochemical properties. Variation of substituent R^2^ (in the case R^1^ and R^3^ = H) demonstrated that compound containing R^2^ = Pr exhibited the best inhibitory activity (IC_50_ of 1.03 μM). Replacing R^3^ in this compound with 2-oxo-2-phenylethyl led to a sharp decrease in inhibitory activity (IC_50_ > 50 μM). Variation of substituent R^1^ (with R^2^ = Ph and R^3^ = H) resulted in changes in inhibitory activity ranging from IC_50_ 1.54–30.71 μM. As a result, it was found that derivative **201a** demonstrated the best efficacy against the full-length human Plk1 in an ELISA assay (Table 6). Furthermore, the MeS-containing prodrug derivatives **202** exhibited potent inhibition of mitotic progression and cellular proliferation, while their metabolic stability was thoroughly evaluated.

### 6.2. [1,2,4]-Triazolo[1,5-a]quinazolines

Book chapter [12] discloses approaches to triazolo[*a*]-annelated quinazolines based on traditional transformation of chloroquinazolines, condensations between quinazoline and azide, copper-catalyzed alkyne-azide cycloaddition reaction, through the coupling of dialkyl/phenyl *N*-cyanoimidocarbonates with the substituted 2-hydrazinobenzoic acids. Some data on biological activity was also provided in the book chapter [12]. The current section of the manuscript contains data on the synthesis and biological activity of triazolo[*a*]quinazolines, which were not included in the book chapter [12] or appeared later.

**Scheme 42:**
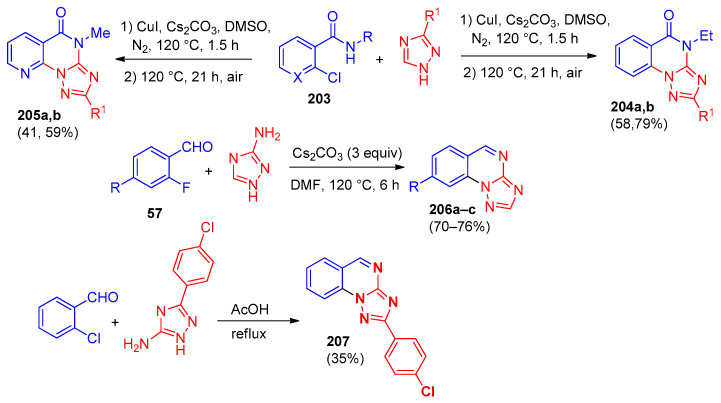
Synthesis of [1,2,4]triazolo[1,5-*a*]quinazolin-5-ones **204** and their aza-analogs **205**, [1,2,4]triazolo[1,5-*a*]quinazolines **206** and **207**. **204**, **205**: R^1^ = H (**a**), Ph (**b**); **206**: R = H (**a**), Br (**b**), Me (**c**).

An approach based on the Ullman coupling of 2-chloroarylamides **203** with triazoles followed by oxidative ring formation was applied for the synthesis of [1,2,4]-triazolo[1,5-*a*]quinazolin-5-ones **204** and their aza-analogs **205** (Scheme 42) [78].

Efficient method was developed for the synthesis of [1,2,4]triazolo[1,5-*a*]quinazolines **206** from 2-fluoro benzaldehydes **57** with 1*H*-1,2,4-triazol-3-amine under metal-free conditions in high yields (Scheme 42) [53]. The process includes an intermolecular condensation followed by metal-free base-promoted intramolecular C–N coupling reaction. It is worth mentioning that the bromine atom stays intact during the reaction and can later be modified or used in additional steps.

2-(4-Chlorophenyl)[1,2,4]triazolo[1,5-*a*]quinazoline **207**, obtained by the reaction of 2-chlorobenzaldehyde with substituted 1*H*-1,2,4-triazol-5-amine (Scheme 42), was evaluated for its in vitro anticancer activity against liver cancer HepG2 and breast cancer MCF7 cell lines, unfortunately, the compound demonstrated low activity [100].

**Scheme 43:**
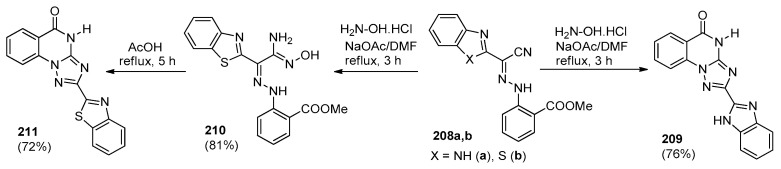
Synthesis of [1,2,4]triazolo[1,5-*a*]quinazolin-5-ones **209** and **211**.

The synthesis of [1,2,4]triazolo[1,5-*a*]quinazolinone derivatives **209** and **211** by reaction of hydrazononitriles with hydroxylamine hydrochloride in presence of sodium acetate was described [101] (Scheme 43). The influence of the nature of the heterocyclic fragment in hydrazononitriles **208** has been demonstrated. Benzimidazolyl-containing hydrazono-nitrile **208a** reacts with hydroxylamine hydrochloride upon boiling in DMF for 3 h leading to triazolo[1,5-*a*]quinazolinone **209**, whereas **208b** containing a benzothiazole fragment under the same conditions forms an amidoxime derivative **210** that did not cyclize even over a longer period (48 h). However, heating compound **210** in glacial acetic acid at reflux yielded the cyclized product **211** in good yield.

**Scheme 44:**
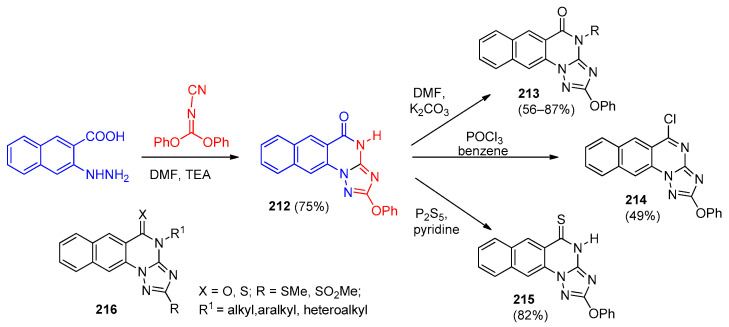
Synthesis of 2-phenoxybenzo[*g*][1,2,4]triazolo[1,5-*a*]quinazolin-5(4*H*)-ones **212**–**215**. **213**: R = Et, allyl, 2(3)-Me-benzyl, 3-OMe-benzyl, 3(4)-CN-benzyl, 4-Cl-benzyl, 4-NO_2_-benzyl, 2-(piperidin-1-yl)ethyl, 2-morpholinoethyl, 2-(phtalimido-2-yl)propyl.

2-Phenoxybenzo[*g*][1,2,4]triazolo[1,5-*a*]quinazolin-5(4*H*)-one **212** was synthesized in good yield by the reaction of diphenyl-*N*-cyanoimidocarbonate with hydrazinonaphthoic acid (Scheme 44) [102]. Alkylation, chlorination and thionation of the lactam group in this compound led to the range of derivatives **213**–**215**. Compounds **212**–**215** were characterized by NMR and HREI-MS analyses.

A series of 2-methylthio-benzo[*g*][1,2,4]triazolo[1,5-*a*]quinazolines **216**, obtained according to Scheme 44, was tested against a variety of Gram-positive bacterial species, Gram-negative bacteria and against ten types of fungi [103]. Most derivatives showed significant antimicrobial activity against six bacterial and six fungal strains. Al-Salahi et al. [104] concluded that the promising compounds could be employed as useful scaffolds for building of new derivatives with more potent antimicrobial effects.

**Figure 12:**
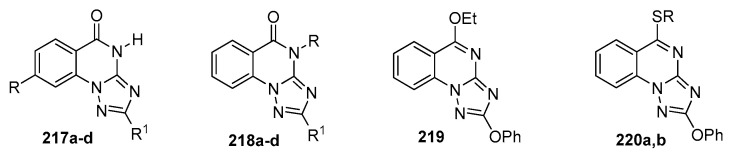
Chemical structures of the compounds **217**–**220**. **217**: R, R^1^ = H, OPh (**a**); H, SMe (**b**); Me, OPh (**c**); H, SO_2_Me (**d**); **218**: R, R^1^ = OPh, 4-NO_2_-Bn (**a**); OPh, Et (**b**); SO_2_Me, allyl (**c**); SO_2_Me, 4-NO_2_-Bn (**d**); **220**: R = Et (**a**), *n*-Pr (**b**).

Various substituted 1,2,4-triazolo[1,5-*a*]quinazolines **217**–**220** (Figure 12) were synthesized **[104]**, and evaluation of their biological effects on heart rate and blood pressure was performed. Compounds **217** were obtained by interaction of the corresponding 2-hydrazino-benzoic acid with diphenyl-*N*-cyanoimidocarabonate, the modification of structure **217** led to triazoloquinazolines **218**–**220**. The structures of derivatives **217b**, **217d** and 2-benzyloxy-1,2,4-triazolo[1,5-*a*]quinazolin-5-one was confirmed later by X-ray data [7,105].

In vivo antihypertensive activity study of the compounds **217**–**220** was performed for rats and mice. The results demonstrated that derivatives **217a**,**c** and **218b** were found to increase the heart rate (33, 14, and 40%, respectively), whereas derivatives **217d** and **218a** decrease the heart rate (23 and 15%, respectively). Moreover, compound **217c** decreases arterial pressure in the rats (6.5 mm H). Derivative **219** has shown to decrease blood pressure (7.8 mm H) and tachycardia effects. Compound **220a** decreases the heart rate (5.9%), at the same time derivative **220b** increases the heart rate (28.5%), both these compounds demonstrate decreasing arterial pressure (6.5 mm H). Al-Salahi et al. [104] suggested that certain compounds could be tested as drugs blocking adrenaline receptors, while others appear to stimulate heart function or could be altered to improve blood pressure reduction.

**Figure 13:**
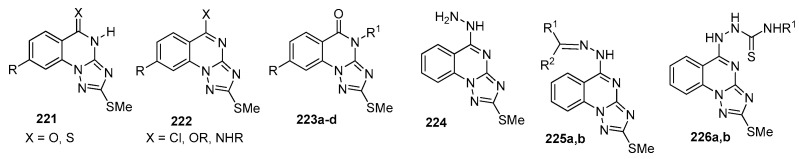
Chemical structures of the compounds **221**, **222**, **223a**–**d**, **224**, **225a**,**b**, **226a**,**b**. **223**: R, R^1^ = H, allyl (**a**); Me, Bn (**b**); H, Bn (**c**); H, 4-NO_2_-Bn (**d**); **225**: R^1^, R^2^ = H, 3-Py (**a**); Me, 4-NO_2_-Ph (**b**); **226**: R^1^ = allyl (**a**), cyclohexyl (**b**).

A series of some previously obtained methylsulfanyl-triazoloquinazoline derivatives **221**–**223** (21 compounds, Figure 13) [106] have been tested against Coxsackievirus B4 and Adenovirus type 7 [107]. The antiviral activity of these compounds against Coxsackie B4 and Adeno type 7 was evaluated in BGM and Hep-2 cell lines. Most tested derivatives were found to possess weak effects on Coxsackie B4 and Adeno type 7, compounds **223** proved to be the most promising. High reduction percentages were observed to viral titers 63.3–83.3% by compounds **223c**, **223b**, and **223d**, which indicates promising effect against Coxsackie B4. The reduction of Adeno type 7 titters were 63.3, 50, and 66.6% demonstrated by **223b**, **223c**, and **223d**, respectively.

Screening and evaluation of antioxidant activity was conducted for the expanded series of 1,2,4-triazolo[1,5-*a*]quinazoline derivatives **221**–**226** (40 compounds) [108]. The antioxidant activity of 1,2,4-triazolo[1,5-*a*]quinazolines was evaluated using the 1,1-diphenyl-2-picryl-hydrazyl (DPPH) reagent. The obtained findings were compared with a standard synthetic antioxidant, 2,6-bis(1,1-dimethylethyl)-4-methylphenol (BHT). All tested compounds exhibited antioxidant activity ranged from weak to moderate and high. It was found that triazoloquinazolines **224**, **225a**,**b**, **226a**,**b** demonstrated the highest capacity to deplete DPPH, and from them, derivatives **224**, **226a**,**b** showed percent inhibitions of 85.9, 91.0, and 91.2, respectively, when compared with BHT (93%).

**Figure 14:**
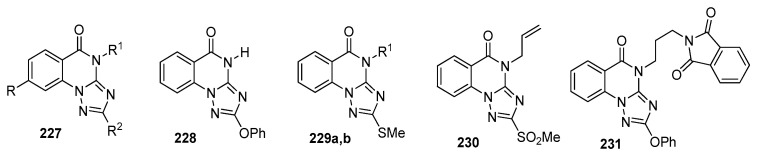
Chemical structures of compounds **227**, **228**, **229a**,**b**, **230**, and **231**. **229**: R^1^ = Et (**a**), allyl (**b**).

Triazoloquinazolines **227** (14 compounds, Figure 14) were reported as a new class of potent α-glucosidase inhibitors [109]. All compounds were synthesized and characterized previously [104,106]. The structure of derivative **229b** was later confirmed by X-ray data [89]. Abuelizz et al. [109] assayed the enzymatic inhibitory activity of compounds **227** against α-glucosidase.

Triazoloquinazolines **227** demonstrated significant activity with the IC_50_ values ranging between 12.70 ± 1.87 and 180.34 ± 1.28 μM. Among them, derivatives **231**, **230**, **229a**, **229b**, and **228** showed the highest inhibitory activity (IC_50_ = 12.70 ± 1.87, 28.54 ± 1.22, 45.65 ± 4.28, 72.28 ± 4.67, and 83.87 ± 5.12 μM, respectively) in relation to that of acarbose (IC_50_ = 143.54 ± 2.08 μM) as a reference drug (Table 6). Molecular modeling study confirmed the importance of binding energy in the stability of complex formed between the docked triazoloquinazolines and the amino acid residues in the active site of the enzyme.

## 7. Conclusions

Looking at current research articles about the discovery of pyrrolo-, indolo-, and azolo[*a*]-quinazoline compounds shows that different types of these molecules have caught researchers’ interest. Although these compounds exhibit markedly distinct structures, most featured molecules remain either unsubstituted at position 4 of the quinazoline nucleus or incorporate oxo-, amino, aryl, or alkoxy substituents.

Considerable prospects are associated with such synthons for [*a*]-annelated quinazolines as 2-(3-butenyl)quinazolin-4(3*H*)-ones, *N*′-phenyl-2-aminobenzohydrazides, 2-halogeno-benzamides, 2-halogenobenzaldehydes, 2-aminobenzonitriles, anthranilamide, isatoic anhydride, 2-hydrazinobenzoic acid, 2-aminoquinazolines, and 2-hydrazinoquinazolines.

A promising line of research is the discovery of new approaches such as cyclocondensations, three-component carbonylative cyclization, Pd-catalyzed domino synthesis or direct C–H arylation, metal-free base-promoted intramolecular C–N coupling reaction. Some green methods were applied, for example, deep eutectic solvent, mechanochemical activation, graphene oxide as mild heterogeneous carbocatalyst, acid functionalized nanoporous silica (SBA-Pr-SO_3_H) as a reusable catalyst.

Noteworthy, many [*a*]-annelated quinazoline derivatives have specific activities in addition to antitumor activity, for example, antibacterial, hypotensive, antidepressant. Pyrazolo[1,5-*a*]quinazolines exhibit anxiolytic, antihyperalgesic and anti-inflammatory activity, whereas pyrido[2′,3′:3,4]pyrazolo[1,5-*a*]quinazoline demonstrate anticancer, antibacterial, and antifungal activity. Isoindolo[*a*]- and imidazo[*a*]-annelated quinazolines show cytotoxic effect, while benzimidazo[1,2-*a*]quinazoline derivatives have been reported as anticancer, antimicrobial, and antitubercular agents. Different [1,2,4]-triazolo[4,3-*a*]quinazolines have proved to exhibit anticancer, antimicrobial, antitubercular, antidepressant, anticonvulsant, antihistaminic, sedative-hypnotic, and vascular-disrupting activities. Some [1,2,4]triazolo[1,5-*a*]quinazoline derivatives demonstrated themselves as antimicrobial, antifungal, hypotensive agents or potent α-glucosidase inhibitors.

We can conclude that quinazolines [4,3-*a*]-annelated by 1,2,4-triazole cycle exhibit more diverse types of biological activity than other quinazolines [*a*]-annelated by azoles.

## Figures and Tables

**Table 1:** Inhibitory activities of compounds **A**, **64p**, **64r**, and **64s** against KDM4D.

Compound	Inhibition Rate at 10 μM	IC_50_ (μM)
**A**	73.33 ± 19.14	3.14 ± 0.18
**64p**	85.06 ± 3.54	4.03 ± 1.02
**64r**	88.82 ± 9.58	0.41 ± 0.03
**64s**	78.06 ± 6.27	1.85 ± 0.14

**Table 2:** BZR Ligand affinity of pyrazolo[1,5-*a*]quinazoline derivatives **72**, **77**, **78** [58].

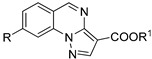			
Compound	R	R^1^	Ki (nM)
**72a**	OMe	Et	102.4 ± 10.4
**72b**	H	Et	529.3 ± 58.8
**77a**	OMe	CH_2_Ph	19.0 ± 1.9
**77b**	OMe	CH_2_-(2-OMe)Ph	0.27 ± 0.04
**77c**	OMe	CH_2_-2-thienyl	16.7 ± 0.9
**77d**	OMe	CH_2_-2-furyl	15.2 ± 1.4
**77e**	OMe	CHMe_2_	97.4 ± 13.8
**78a**	H	CH_2_Ph	25.9 ± 1.98
**78b**	H	CH_2_-(2-OMe)Ph	3.16 ± 0.54
**78c**	H	CH_2_-2-thienyl	13.2 ± 0.7

**Table 3:** Binding at GABA_A_-R subtype of compounds **89**–**94**, **96** [61].

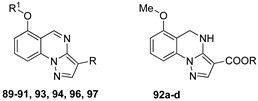			
Compound	R	R^1^	I, %
**89a**	CN	H	2
**89b**	COOEt	H	32
**90a**	CN	CH_2_Ph	5
**90b**	COOEt	CH_3_	19
**91a**	COOCH_2_Ph	CH_3_	14
**91b**	COOCH_2_(2-OCH_3_Ph)	CH_3_	18
**91c**	COOCH_2_-2-furyl	CH_3_	10
**92a**	COOCH_2_Ph	-	14
**92b**	COOCH_2_(2-OCH_3_Ph)	-	14
**92c**	COOCH_2_-2-furyl	-	7
**92d**	COOEt	-	1
**93**	1,2,4-oxadiazol-3-yl-5-methyl	CH_2_Ph	10
**94a**	CONH_2_	H	5
**94b**	CONH_2_	CH_2_Ph	5
**96a**	1,2,4-triazol-3-yl	CH_3_	9
**96b**	1,2,4-triazol-3-yl	CH_2_Ph	9
**97**	I	CH_2_Ph	43.7

**Table 4:** Antibacterial activity of pyrido[2′,3′:3,4]pyrazolo[1,5-*a*]quinazoline derivatives **130**–**132** (minimum inhibitory concentration MIC, μg/mL) [69].

Compound	*Staphylococcus* *Aureus* MTCC96	*Bacillus Subtilis* MTCC121	*Staphylococcus Aureus* MLS162940	*Micrococcus Luteus* 2470MTCC	*Klebsiella Planticola* MTCC530
**130a**	3.9	3.9	7.8	3.9	3.9
**130b**	>125	15.6	>125	>125	>125
**130c**	3.9	3.9	3.9	3.9	3.9
**130f**	3.9	3.9	7.8	3.9	>125
**131a**	3.9	3.9	15.6	7.8	3.9
**132c**	3.9	3.9	7.8	>125	>125
**132d**	>125	>125	>125	>125	7.8
**132e**	15.6	15.6	>125	>125	>125
**132f**	3.9	3.9	7.8	7.8	7.8
**132h**	3.9	3.9	7.8	>125	7.8
Ciprofloxacin	3.9	3.9	3.9	3.9	3.9

**Table 5:** Inhibition rate of compounds **161** and **164** on melanoma cells A375 in vitro at 100 μM [81].

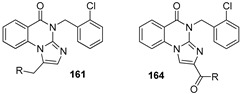					
Compound	R	% Inhibition	Compound	R	% Inhibition
**161**	4-Methylpiperazin-1-yl	76.15	164	(3-MeOC_6_H_4_)NH	24.42
**164**	3-(OCF_3_)C_6_H_4_NH	24.08	164	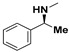	21.89
**164**	3-CF_3_C_6_H_4_NH	27.93	164	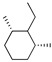	24.77
SHP244		13.81	Sorafenib		14.79

**Table 6:** Medicinally important [1,2,4]-triazolo[4,3-*a*]quinazolines.

Compound	Substituents	Biological Activity	LC_50_ Value	Reference
**175**	R = 4-Cl	Anticonvulsant	88.02 mg/kg	[85]
**175**	R = 4-Br	Anticonvulsant	94.60 mg/kg	[85]
**176**	R = 4-Me	Antidepressant	Percentage decrease in immobility duration 82.69% at a dose of 50 mg/kg	[86]
**193**	Ar = 4-MeOC_6_H_4_	Inhibitory activity against SHP2 protease	Percentage inhibition 28.20% at 100 μM	[81]
**201**	R^1^ = R^3^ = H, R^2^ = Pr	PBD of Plk1 Inhibitory	1.03 μM	[88]
**231**		α-Glucosidase inhibitor	12.70 μM	[89]

## Data Availability

The raw data supporting the conclusions of this article will be made available by the authors on request.

## Abbreviations

The following abbreviations are used in this manuscript:

TFA	Trifluoroacetic acid
CPT	Camptothecin
NIS	N-iodosuccinimide
NBS	*N*-bromosuccinimide
NOE	Nuclear Overhauser effect
DMSO	Dimethyl sulfoxide
TBAB	Tetrabutylammonium bromide
PEG	Polyethylene glycol
TFE	2,2,2-Trifluoroethanol
LAG	Liquid-assisted grinding
HTS	High-throughput screening
DMF	*N*,*N*-Dimethylformamide
GABA	Gamma-aminobutyric acid
HCA	Hierarchical Cluster Analysis
ERK	Extracellular signal-regulated kinase
GBBR	Groebke–Blackburn–Bienaymé reaction
MES	Maximal electroshock
ATP	Adenosine triphosphate
SAR	Structure–activity relationship
DFT	Density Functional Theory
FMO	Frontier molecular orbital
TfOH	Trifluoromethanesulfonic acid
PTSA, TsOH	*p*-Toluenesulfonic acid
DCM	Dichloromethane
DCE	1,2-Dichloroethane
NMR	Nuclear magnetic resonance
MsOH	Methanesulfonic acid
DMF-DtBA	Dimethylformamide-di-*tert*-butylacetate
PIFA	(Bis(trifluoroacetoxy)iodo)benzene
DABCO	1,4-diazabicyclo[2.2.2]octane
BOP	Benzotriazol-1-yloxy-tris(dimethylamino)phosphonium hexafluorophosphate
DPPH	1,1-diphenyl-2-picryl-hydrazyl
MOF	Metal–organic frameworks
NCI	National Cancer Institute
HDAC	Histone deacetylase
VEGFR	Vascular endothelial growth factor receptor
HREI-MS	High-resolution electron ionization mass spectrometry
TNF	Tumor Necrosis Factor
TLC	Thin-layer chromatography
